# Time-Resolved, In Situ DRIFTS/EDE/MS Studies on Alumina-Supported Rhodium Catalysts: Effects of Ceriation and Zirconiation on Rhodium–CO Interactions**

**DOI:** 10.1002/cphc.201402122

**Published:** 2014-07-18

**Authors:** Anna B Kroner, Mark A Newton, Moniek Tromp, Otello M Roscioni, Andrea E Russell, Andrew J Dent, Carmelo Prestipino, John Evans

**Affiliations:** [a] Diamond Light Source, Diamond House, Harwell Science and Innovation Campus Chilton, Oxfordshire, OX11 0DE (UK); [b] The European Synchrotron Radiation Facility 71 Rue des Martyrs, Grenoble, 38043 (France) E-mail: newton@esrf.fr; [c] Technische Universität München Lichtenbergstrasse 4, 85748 Garching (Germany); [d] School of Chemistry, University of Southampton Highfield, Southampton, SO17 1BJ (UK) E-mail: je@soton.ac.uk; [e] Institut des Sciences Chimiques de Rennes, Université de Rennes 1 35042 Rennes Cedex (France); [f] Research Complex at Harwell, Rutherford Appleton Laboratory Didcot, OX11 1FA (UK)

**Keywords:** analytical methods, cerium, rhodium, supported catalysts, zirconium

## Abstract

The effects of ceria and zirconia on the structure–function properties of supported rhodium catalysts (1.6 and 4 wt % Rh/γ-Al_2_O_3_) during CO exposure are described. Ceria and zirconia are introduced through two preparation methods: 1) ceria is deposited on γ-Al_2_O_3_ from [Ce(acac)_3_] and rhodium metal is subsequently added, and 2) through the controlled surface modification (CSM) technique, which involves the decomposition of [M(acac)_*x*_] (M=Ce, *x*=3; M=Zr, *x*=4) on Rh/γ-Al_2_O_3_. The structure–function correlations of ceria and/or zirconia-doped rhodium catalysts are investigated by diffuse reflectance infrared Fourier-transform spectroscopy/energy-dispersive extended X-ray absorption spectroscopy/mass spectrometry (DRIFTS/EDE/MS) under time-resolved, in situ conditions. CeO_*x*_ and ZrO_2_ facilitate the protection of Rh particles against extensive oxidation in air and CO. Larger Rh core particles of ceriated and zirconiated Rh catalysts prepared by CSM are observed and compared with Rh/γ-Al_2_O_3_ samples, whereas supported Rh particles are easily disrupted by CO forming mononuclear Rh geminal dicarbonyl species. DRIFTS results indicate that, through the interaction of CO with ceriated Rh particles, a significantly larger amount of linear CO species form; this suggests the predominance of a metallic Rh phase.

## 1. Introduction

The interaction of CO with rhodium-containing catalysts has been intensively studied in recent decades.[1–7] This interest stems largely from the use of rhodium in three-way automotive exhaust catalysts (TWC). In addition to the metal components of modern TWCs, a complex mixture of precious-metal particles (Rh, Pt, Pd) and various oxide materials are included in the catalysts’ formulation. Amongst these, cerium oxide has emerged as a highly important promoter material.[8, 9] The addition of ceria (CeO_2_) to the (Al_2_O_3_-based) supports greatly enhances catalyst performance, in part because of its ability to store and release oxygen. Moreover, ceria has been also replaced by ceria–zirconia, which has even higher oxygen storage properties plus improved thermal stability.[10–12] Rhodium is included in the formulation of such catalysts due to its superior ability to catalyse the reduction of NO in reducing atmospheres containing hydrocarbons. Furthermore, the unique coordination chemistry of rhodium gives rise to a large variety of carbonyl and nitrosyl chemisorbed species.[4, 13] Indeed, this propensity is also exploited in solution- and vapour-phase carbonylation chemistry.[14]

How this “organometallic” aspect of the behaviour of rhodium might transfer and complete with “extended metal surface” type behaviour within, for instance, the TWC paradigm, is an intriguing question. Carbonyl species chemisorbed on metal particles or surfaces are sensitive probes of the character of supported rhodium catalysts. As established in the pioneering work of Yang and Garland,[7] the three typical forms of CO chemisorbed on Rh are the geminal dicarbonyl, Rh(CO)_2_, species (identified by the *ν*(s) and *ν*(as) M_C–O_ IR modes); the linearly bonded form; and the bridged form. It has also been demonstrated that, under certain conditions, a number of other minor species are formed. For example, upon oxidation, a new *ν*(CO) band is observed at 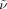
=2127 cm^−1^, which can be assigned to a RhO(CO) site.[15, 16] It has been suggested that irregular and well-dispersed Rh particles favour the formation of Rh(CO)_2_ sites, whereas extended surfaces and larger Rh particles favour the linear and bridged CO forms.[17] For instance, according to Hyde et al.,[18] dicarbonyl species are formed as isolated surface organometallic species or at edge sites. Linear and bridged species are suggested to form on metallic Rh sites in larger crystallites (Rh^0^_*x*_),[17, 19] as demonstrated by IR studies on both powder catalysts and extended, low index, Rh surfaces. A number of parameters have considerable bearing on the extent to which Rh(CO)_2_ centres may be formed from Rh nanoparticles: 1) the average size of the Rh particles, and 2) the presence of Cl in the catalysts. Joyner and Johnston provided convincing evidence that Cl promoted the formation of Rh(CO)_2_ sites when the average Rh particle size in the catalysts was similar.[20] It is also clear that this site can be formed in chlorine-free materials.[21] Moreover, it has been shown that the presence of gas-phase oxidants, such as NO/O_2_, enhanced dramatically the rate of the oxidative disruption of Rh particles to form Rh *gem*-dicarbonyl species.[22, 23] The CO dissociation process taking place on relatively small Rh nanoparticles (less than 3 nm) is also a source of the oxidative disruption of Rh particles.[24, 25]

Because modern TWCs are composed of precious-metal particles (e.g. Rh, Pt, Pd) and various oxide materials, such as CeO_*x*_ and ZrO_2_, typically supported on Al_2_O_3_,[8, 9] it is important to understand the effects of these components on CO interactions. This study is focussed on 4 and 1.6 wt % Rh-based materials supported on Al_2_O_3_ and doped with CeO_*x*_ and/or ZrO_2_ prepared from β-diketonate precursors, which we have shown to afford a close association between Rh and these oxides.[22] The structural changes of the metal sites induced by CO adsorption have been monitored in a time-resolved manner by the combined techniques of energy-dispersive extended X-ray absorption spectroscopy (EDE), diffuse reflectance infrared Fourier-transform spectroscopy (DRIFTS) and mass spectrometry (MS).[21, 23] EDE is a unique type of X-ray absorption spectroscopy (XAS) measurement that provides the band of energies for the X-ray absorption fine structure (XAFS) spectrum in milliseconds. Simultaneous monitoring with DRIFTS (data acquisition of 100 ms) and MS techniques gives an invaluable insight into the catalytic systems under operating conditions. These dynamic, time-resolved studies can be applied to track the structural and kinetic changes and identify any transient species. However, the signal-to-noise ratio is considerably higher when compared with standard XAS measurements, which were also applied in this research. In the scanning XAS measurements, a sample is studied under selected conditions to provide direct, more accurate structural information about the catalytic system in a relevant environment; however, only under steady-state conditions.

## 2. Results and Discussion

CO interactions with Rh catalysts have been studied in situ in the DRIFTS cell by two different experiments: time-resolved studies by means of combined EDE/DRIFTS/MS apparatus[24] at ID24 of the European Synchrotron Radiation Facility (ESRF) and steady-state measurements by means of a scanning XAS technique combined with MS at BM29 at the ESRF. For both experiments, the catalyst is pre-treated in situ before the reaction by heating up to 573 K under a flow of 5 % H_2_/He, then oxidised by 5 % O_2_/He until H_2_O and carbonaceous deposition disappear from the system, and subsequently the gas is switched back to 5 % H_2_/He. After full pre-treatment, the system was cooled under H_2_ to the working temperature and purged with He. Throughout the time-resolved experiment, the sample was exposed to 5 % CO/He (25 mL min^−1^) for 60 s and the flowing gas was subsequently switched to He for 60 s. The Rh–CO interactions were studied at 323, 373, 423, 473 and 573 K. Each experiment lasted 120 s and the EDE/DRIFTS/MS data were collected simultaneously. A new sample was loaded for each experiment at the selected temperature.

In the case of the steady-state experiment, after the pre-treatment stage, a sample was exposed to 5 % CO/He for 30 min until the XAS spectrum was recorded. The XAS measurements were acquired at the same time as the switch to the CO/He gas feedstock occurred. Then the catalyst was heated to 423 and 573 K under a flow of continuous CO/He. The spectra were collected at 323 K under He, after cleaning, and then during purging with 5 % CO/He at 323, 423 and 573 K.

### 2.1. Rh/Al_2_O_3_

**Figure 1:**
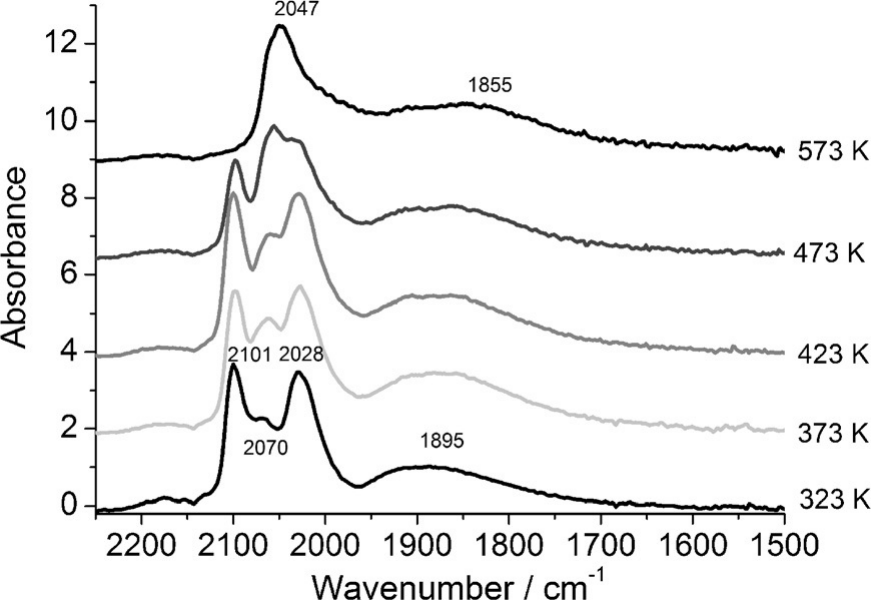
DRIFTS spectra of 4 wt % Rh/Al_2_O_3_ after CO exposure at 323, 373, 423, 473 and 573 K (4 spectra averaged at 59 s of CO exposure).

The DRIFTS spectra of 4 wt % Rh/γ-Al_2_O_3_ during CO exposure at different temperatures (Figure 1) show that, in the range of 323–473 K, all spectra displayed geminal dicarbonyl species [Rh^I^–(CO)_2_], which could be identified by the symmetric (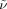
≈2101 cm^−1^) and asymmetric (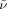
=2028 cm^−1^) *ν*(C–O) bands.[7] These bands overlap with the vibration of the linear species (Rh–CO) at 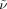
=2070 (323 K) to 2057 cm^−1^ (473 K).[17] The bridged CO species (Rh_*n*_–(CO), *n*≥2) afforded a broad peak (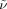
≈1883–1895 cm^−1^).[16] The spectrum of Rh^I^(CO)_2_ showed no significant shift in wavenumber. At higher temperatures (473–573 K), the proportion of the geminal dicarbonyl was reduced with only linear and bridged CO species on a Rh surface observed at 573 K; this suggested the formation of extended Rh surfaces. A small redshift of the symmetric stretching peak for the dicarbonyl species from 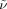
=2101 cm^−1^ at 323 K to 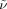
=2097 cm^−1^ at 473 K was observed; this may be due to the increase in electrodynamic coupling between the dynamic moment of the adsorbed complex and the alumina substrate.[26] The redshifts observed for the linear CO species at higher temperatures (from 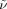
=2070 cm^−1^ at 323 K through 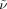
≈2059 cm^−1^ at 423 K, to 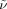
≈2047 cm^−1^ at 573 K) and for the broad peak for bridged CO (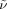
≈1895 cm^−1^ at 323 K, 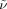
≈1883 cm^−1^ at 423 K, 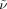
≈1855 cm^−1^ at 573 K) is typical of a gradual reduction of the surface coverage of these species.

**Figure 2:**
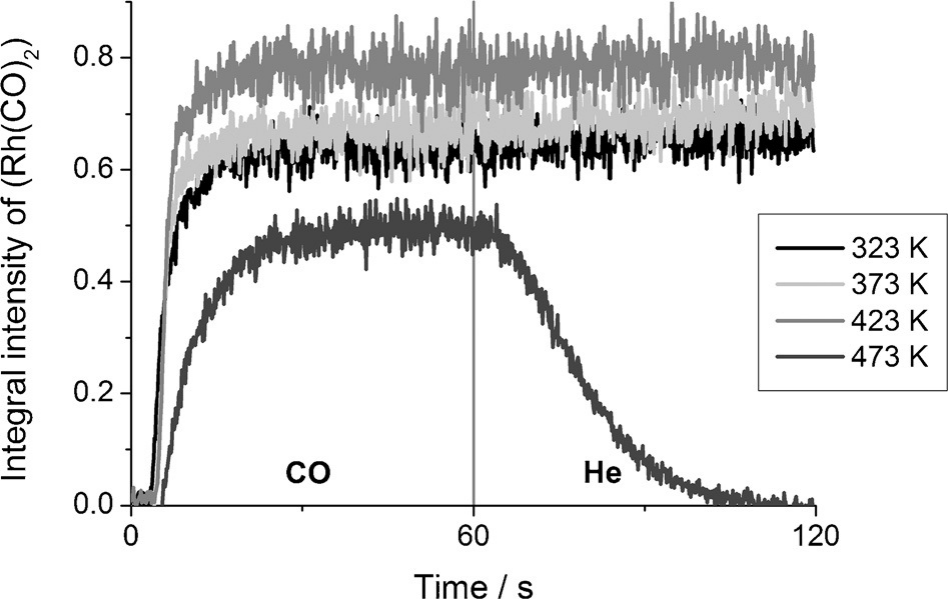
The evolution of the integral intensity of *ν*(CO)(s) in Rh^I^(CO)_2_ at different temperatures.

The integral intensity of the *ν*(CO) bands of Rh^I^(CO)_2_/γ-Al_2_O_3_ evolved at a rate that was independent of the temperature (Figure 2) and stabilised in about 20 s for measurements conducted up to 423 K. The relative increase in the integral intensity at times longer than 20 s observed upon increasing the temperature from 323 to 423 K indicated more extensive oxidative disruption of the Rh overlayer to isolated Rh(CO)_2_ at higher temperature. Furthermore, no decomposition of CO from the geminal dicarbonyl species was evident when the gas feed was switched to He. However, at 473 K, the amount of Rh^I^(CO)_2_/γ-Al_2_O_3_ formed decreased and desorption of these species was observed during He exposure. After 40 s of He flow, the Rh(CO)_2_ bands were completely lost. However, the IR spectra showed that the other adsorbed linear and bridged CO species did not desorb from the Rh surface throughout He exposure (Figure S1 in the Supporting Information). These results indicate that the mononuclear Rh^I^(CO)_2_ sites on γ-Al_2_O_3_ are unstable above about 423 K.

Table 1 presents the structural and statistical parameters derived from the analyses of the Rh K-edge extended X-ray absorption fine structure (EXAFS) for 4 wt % Rh/Al_2_O_3_ before and after 5 % CO/He exposure in the temperature range of 323–573 K. The EDE spectra and their Fourier transforms are included in Figure S2 in the Supporting Information; changes in CN as a function of temperature are presented in Figure S3 in the Supporting Information. The refined parameters from the EXAFS analysis show that, within the timescales of this experiment (≈10 times 100 ms), structural changes accompany the exposure of the catalyst to a flow of 5 % CO/He. The CN of the Rh–Rh shell decreased from about 7.2 under H_2_ to about 5.4 after CO exposure for 59 s at 323 K. The Rh–Rh bond length changed throughout this CO exposure from 2.64 to about 2.68 Å. Similar results were observed for catalysts at increased temperatures up to 423 K.

**Table 1:** Structural and statistical data of the Rh–Rh shell derived from the analysis of Rh K-edge EXAFS over 4 wt % Rh/Al_2_O_3_ at temperatures and conditions indicated (EDE measurements)^[a]^

Conditions	CN^[b]^	*r* [Å]	DW^[c]^ [Å^2^]	*R* [%]
5 % H_2_/He at 323 K	7.2(4)	2.64(1)	0.0115	52
5 % CO/He at 323 K	5.4(3)	2.68(1)	0.0115	53
5 % H_2_/He at 373 K	8.3(5)	2.65(1)	0.0120	47
5 % CO/He at 373 K	6.3(4)	2.68(1)	0.0120	53
5 % H_2_/He at 423 K	7.6(4)	2.64(1)	0.0135	54
5 % CO/He at 423 K	4.5(4)	2.69(1)	0.0135	59
5 % H_2_/He at 473 K	6.6(5)	2.64(1)	0.0150	58
5 % CO/He at 473 K	6.1(5)	2.67(1)	0.0150	52
5 % H_2_/He at 573 K	7.3(6)	2.64(1)	0.0175	56
5 % CO/He at 573 K	6.3(6)	2.66(1)	0.0175	56

[a] The data range used was 3–11 Å^−1^; the *R* fitting range was 1–6 Å; the constant amplitude fitting parameter (AFAC)=1. Values in parentheses are statistical errors generated in the EXCURV98 program. [b] CN=coordination number. [c] DW=Debye–Waller factor=2*σ*^2^.

The structural changes observed for the 4 wt % Rh/Al_2_O_3_ catalysts are in good agreement with the DRIFTS results; this highlights the larger oxidative disruption of Rh particles with increasing temperature up to 423 K. In the range of 473–573 K, there is no significant change in the EXAFS after CO exposure (60 s); the N_1_^Rh^ value decreases from about seven under H_2_ to about six. These results suggest that the Rh particles are quickly reduced under CO exposure at 573 K and CO adsorbs on the metallic Rh surface in linear and bridging sites. The formation of these species at the expense of geminal dicarbonyl species after the heating of Rh catalysts in the CO flow has been previously reported by others, and is indicative of the agglomeration of isolated rhodium ions and the formation of metal crystallites.[4, 27, 28]

CO interactions for the 4 wt % Rh/γ-Al_2_O_3_ catalysts were also investigated by scanning Rh K-edge XAFS to obtain more accurate structural parameters and to investigate the presence of other neighbouring atoms. The structural parameters obtained for the Rh K-edge *k*^3^-weighted EXAFS analyses (Table 2) used a fixed DW factor for the first Rh–Rh shell, as established by Jyoti;[29] those for further Rh–Rh shells were refined by minimising the *R* factor. The Rh–Rh bond length of 2.67 Å, with a Rh–Rh CN of about 6.8, was in good agreement with the values obtained from the EDE data (CN_Rh–Rh_≈7.2) under 5 % H_2_/He at 323 K (Table 1). However, the longer acquisition time of the EXAFS spectrum (≈30 min) allowed the detection of the non-bonded Rh–Rh shells: 1.7 at 3.77 Å, 4.7 at 4.64 Å and 3.6 at 5.24 Å; these corresponded to a fragment of the bulk face-centred cubic (fcc) structure.[30] After 30 min of CO exposure at 323 K, only minor structural changes were evident: CN(Rh–Rh) decreased from about 6.8 under H_2_ to about 5.3 under CO. At the same time, a small expansion in the Rh–Rh bond length (from 2.67 to 2.69 Å) was also observed. Increasing the temperature to 423 K did not cause any further observable structural changes. However, at 573 K, disruption of the Rh particles was seen, as marked by a decrease in both the CN_Rh–Rh_ and first-shell Rh–Rh bond length. Even after long CO exposure at increased temperature, the Rh particles of 4 wt % Rh/Al_2_O_3_ still contained further Rh–Rh shells that were typical of the second, third and fourth shells of a fcc metallic structure.

**Table 2:** Structural and statistical data derived from EXAFS analyses of 4 wt % Rh/Al_2_O_3_ at temperatures and conditions indicated (scanning EXAFS measurements)^[a]^

Conditions	CN^[b]^	*R* [Å]	DW [Å^2^]	*R* [%]
5 % H_2_/He, 323 K	6.8(2) 1.7(2) 4.7(1) 3.6(2)	2.674(4) 3.773(3) 4.645(4) 5.242(5)	0.011 0.011 0.011 0.012	30
5 % CO/He, 323 K	5.3(2) 1.4(1) 3.4(2) 3.5(2)	2.689(5) 3.819(4) 4.676(3) 5.293(4)	0.012 0.012 0.012 0.012	24
5 % CO/He, 423 K	5.7(3) 1.4(2) 3.0(3) 3.5(2)	2.684(5) 3.786(3) 4.666(4) 5.274(3)	0.014 0.016 0.014 0.015	21
5 % CO/He, 573 K	4.7(3) 0.8(2) 1.2(3) 1.9(2)	2.667(5) 3.739(4) 4.652(3) 5.265(3)	0.018 0.018 0.017 0.017	36

[a] Data range used was 3.3–16 Å^−1^; *R* fitting range was 1–6 Å; AFAC=1. Values in parentheses are statistical errors generated in the EXCURV98 program. [b] Scatterer refined values are those of the Rh–Rh shells only.

Analogous experiments performed with a lower metal loading, 1.6 wt % Rh/γ-Al_2_O_3_, catalyst showed that the adsorption characteristics and overall processes involved were similar. The IR (Figure S4 in the Supporting Information) and EDE (Figures S5 and S6 and Table S1 in the Supporting Information) results demonstrated the oxidative disruption of rhodium nanoparticles on the alumina support. All three IR-active CO species were formed during CO exposure, as in the case of the 4 wt % Rh sample. However, a larger fraction of CO was adsorbed as the Rh geminal dicarbonyl species, relative to 4 wt % Rh/γ-Al_2_O_3_. At 323 K, the decrease in the Rh–Rh CN of 1.6 wt % Rh/Al_2_O_3_ reflects a decrease in the number of atoms in the average particle from about 13 to 7. Moreover, an additional RhO shell was detected. A further oxidative disruption of Rh particles can be observed when increasing the temperature, with a more dominant Rh–O contribution (Figure S7 in the Supporting Information). Because Rh–Rh bonding was still present after 60 s of CO exposure and CO sites on the metallic Rh were observed, these results indicate that there was only partial disruption of the Rh particles to atomically dispersed Rh species. Theoretical calculations showed that surface Rh^I^(CO)_2_/γ-Al_2_O_3_ sites originated from the oxidative disruption of Rh^0^ crystallites mediated by surface hydroxyl groups, giving rise to a reconstruction of the support surface to accommodate stable, square-planar Rh^I^ centres.[30]

Combined DRIFTS/EDE studies showed that the same CO species were adsorbed on the Rh surface (geminal dicarbonyl, linear and bridged CO species) for both Rh catalysts investigated (4 and 1.6 wt % Rh), with a relatively larger fraction of Rh^I^(CO)_2_ present for the sample with a lower concentration of Rh. As expected, we observed a larger oxidative disruption of Rh particles for 1.6 wt % Rh/Al_2_O_3_; the Rh–Rh bond length did not change after CO exposure. On the other hand, an increase in the Rh–Rh bond length after CO exposure was observed for the catalyst with a higher Rh loading in the temperature range studied from 323 to 473 K. The phenomenon of CO adsorption is consistent with the mechanism suggested by Basu et al.,[2] wherein the adsorption of CO on Rh^0^ surface sites weakens the Rh–Rh bonds and forms smaller Rh particles across the whole support. Such disruption in favourable sites leads to the liberation of atomically dispersed rhodium species that can migrate across the oxide surface to isolated hydroxyl groups, creating geminal dicarbonyl species. The linear and bridged species adsorbed on the extended surface area seem to be stable. At higher temperature, 473–573 K, another effect of adsorbed CO comes into prominence, which leads to the formation of the metallic Rh fraction at the expense of isolated Rh sites.

### 2.2. Rh/CeO_*x*_/Al_2_O_3_

The effect of ceria on the catalytic performance of rhodium catalysts was studied with ceriated rhodium catalysts derived from two different procedures. In the first method, ceria was deposited on the alumina support, and subsequently, rhodium metal was added (method I); the second procedure followed the controlled surface modification (CSM) method, in where ceria was added onto the pre-formed Rh/γ-Al_2_O_3_ metal (method II).[22]

**Figure 3:**
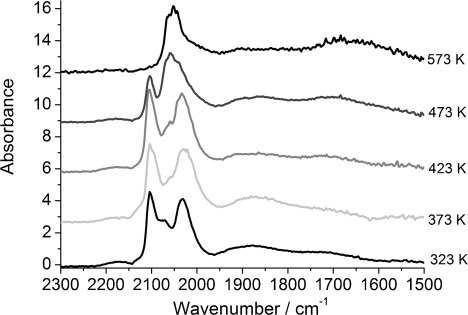
DRIFTS spectra of 4 wt % Rh/CeO_*x*_/Al_2_O_3_ (method I) after CO exposure at 323, 373, 423, 473 and 573 K.

The DRIFTS spectra of 4 wt % Rh/CeO_*x*_/Al_2_O_3_ (method I) after 59 s of 5 % CO/He exposure (Figure 3), similar to that for 4 wt % Rh/Al_2_O_3_, display two sharp peaks at 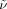
=2102 and 2031 cm^−1^ assigned to [Rh^I^(CO)_2_], in the temperature range 323–423 K, analogously to 4 wt % Rh/Al_2_O_3_. A sharp band of linearly bonded CO (Rh–CO) is observed at 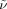
=2073 and 2059 cm^−1^ at 323 and 573 K, respectively. As for 4 wt % Rh/Al_2_O_3_, a redshift was observed for the linear species from 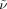
=2073 to 2053 cm^−1^ and for the bridged species from 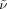
=1875 to 1866 cm^−1^ with increasing temperature. The only apparent difference between 4 wt % Rh/Al_2_O_3_ and 4 wt % Rh/CeO_*x*_/Al_2_O_3_ (method I) was the presence of an additional peak at 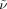
≈1720 cm^−1^ at 323 K, which was redshifted to 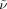
=1670 cm^−1^ with increasing temperature.

**Figure 4:**
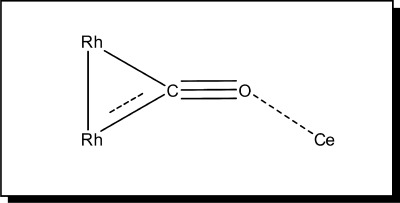
The bridged CO species between Rh and Ce atoms.

Based on a comparison with the IR spectra of known metal carbonyl compounds,[31, 32] the peak centred at 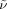
=1720 cm^−1^ at 323 K might be ascribed to a Rh–CO site, in which the carbonyl oxygen atom interacts with a cerium cation of the support (Figure 4). In an IR spectroscopy study of CO interactions with Rh/CeO_2_/SiO_2_, Kiennemann et al. observed this characteristic peak of CO at 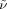
=1725 cm^−1^.[31] Furthermore, in a study of CO and NO interactions with Rh/Pt/CeO_2_/Al_2_O_3_ catalysts by temperature-programmed desorption, Loof et al. showed that reduced ceria strongly influenced both CO and NO bonding and desorption mechanisms on Pt and Rh.[33]

**Figure 5:**
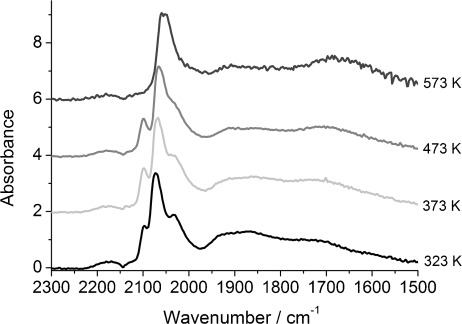
DRIFTS spectra of 4 wt % Rh/CeO_*x*_/Al_2_O_3_ (method II) after CO exposure at 323, 373, 473 and 573 K.

In contrast, the DRIFTS spectra of 4 wt % Rh/CeO_*x*_/Al_2_O_3_ prepared by the CSM (method II) after 59 s of 5 % CO/He exposure (Figure 5) in the temperature range of 323–473 K showed mainly a sharp peak appearing at 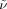
≈2070 cm^−1^, which was assigned to linear CO species, the intensity of which was larger than the residual CO peaks present on other Rh catalysts. The bridged CO species gave a broad feature at 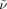
≈1889 cm^−1^. Two shoulders associated with rhodium geminal dicarbonyl species at 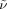
=2098 and 2030 cm^−1^ were also observed. The broad signal at 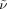
≈1725 cm^−1^, which was characteristic of bridged CO species between 2 or 3 Rh atoms and a Ce atom (Figure 4), was redshifted to 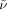
=1685 cm^−1^ at 573 K. After increasing the temperature to 573 K, a redshift from 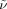
=2073 to 2056 cm^−1^ was observed for the linear species, with a blueshift for the bridged species from 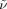
=1889 to 1900 cm^−1^. Others have associated such shifts with an increase in the Rh particle size upon which the bridged CO species is adsorbed.[3] Furthermore, no trace of Rh^I^(CO)_2_ species was evident at 573 K, as for all the other Rh catalysts.

Evidently, the synthetic method has an effect on the “Rh–CO” centres formed during CO exposure. Preparation method I, wherein rhodium is supported on ceria–alumina, does not seem to significantly change the adsorption of rhodium. At lower temperatures, the pronounced absorption bands associated with Rh^I^(CO)_2_ species indicate that strong Rh bonding with O from the alumina support is retained. However, in the case for which ceria is deposited on a pre-supported Rh catalyst (method II), an apparent difference in structural behaviour is observed. The obtained results suggest that ceria is in close vicinity to rhodium particles and can be as a ceria “corral”, which keeps rhodium particles in a metallic form, as evidenced by the presence of mostly linear and bridged CO species mostly on the rhodium surface.

**Figure 6:**
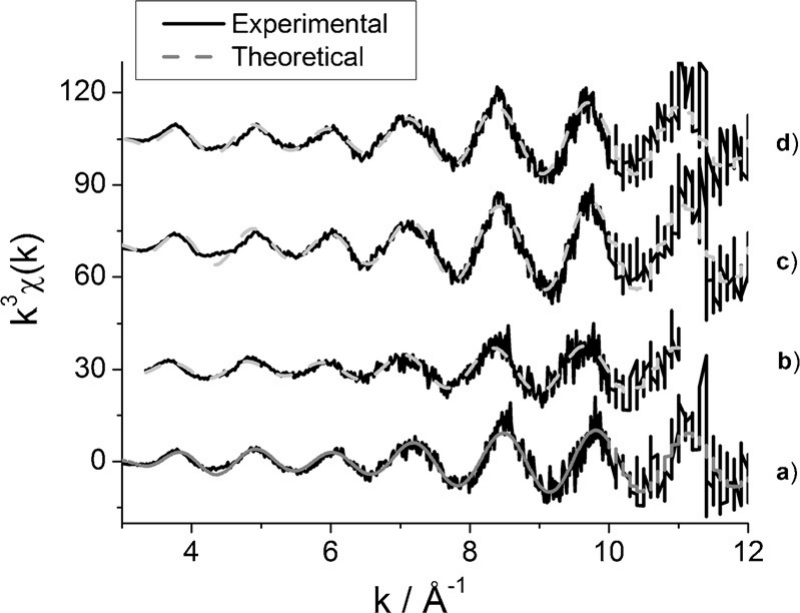
The *k*^3^-weighted Rh K-edge EDE data obtained for 4 wt % Rh/CeO_*x*_/Al_2_O_3_ (method I) under a) 5 % H_2_/He and b) 5 % CO/He (average of 10 spectra taken at 59 s), and for 4 wt % Rh/CeO_*x*_/Al_2_O_3_ (method II) under c) 5 % H_2_/He and d) 5 % CO/He at 323 K.

**Figure 7:**
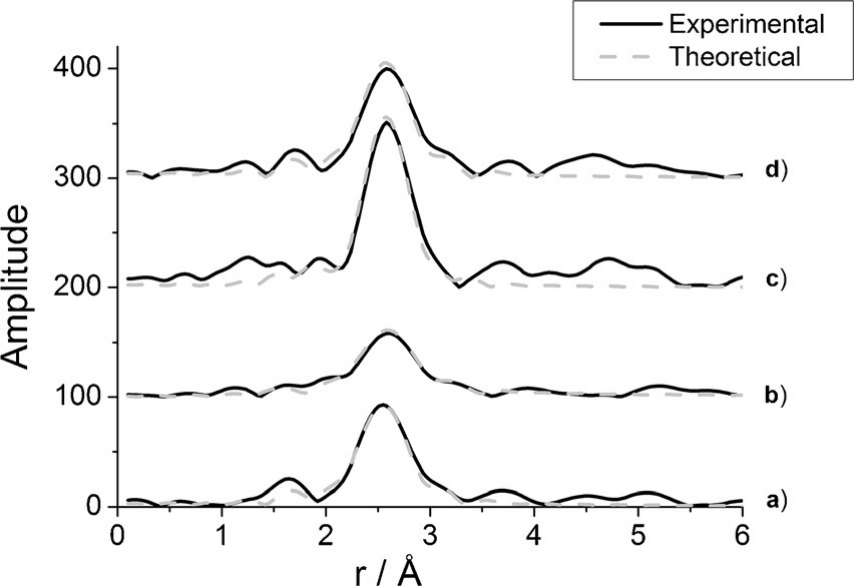
Fourier transform of *k*^3^-weighted Rh K-edge EDE data obtained for 4 wt % Rh/CeO_*x*_/Al_2_O_3_ (method I) under a) 5 % H_2_/He and b) 5 % CO/He (average of 10 spectra taken at 59 s), and for 4 wt % Rh/CeO_*x*_/Al_2_O_3_ (method II) under c) 5 % H_2_/He and d) 5 % CO/He at 323 K.

EDE measurements for ceriated rhodium catalysts performed simultaneously with DRIFTS measurements showed that some structural changes occurred in rhodium particles when the samples were exposed to the flow of CO/He gas feedstock. Figures 6 and 7 show *k*^3^-weighted EDE spectra and their Fourier transforms, respectively, for both ceriated 4 wt % Rh catalysts before and after 5 % CO/He exposure at 323 K (analyses are given in Tables 3 and 4). Rhodium K-edge EXAFS analysis was performed at the start and at the end of CO exposure to study the disruptive effect of CO molecules towards Rh particles of a variety of Rh catalysts studied. For each of the spectral analyses, ten EDE experimental spectra were taken at the beginning and over the final second of CO exposure and averaged for the EXAFS refinement.

**Table 3:** Structural and statistical data for the Rh–Rh shell derived from the analysis of Rh K-edge EXAFS over 4 wt % Rh/CeOx/Al_2_O_3_ (method I) at temperatures and conditions indicated^[a]^

Conditions	CN	*r* [Å]	DW [Å^2^]	*R* [%]
5 % H_2_/He, 323 K	5.7(3)	2.65(1)	0.0115	51
5 % CO/He, 323 K	4.4(3)	2.69(1)	0.0115	53
5 % H_2_/He, 373 K	5.3(4)	2.64(1)	0.0120	61
5 % CO/He, 373 K	3.3(2)	2.67(1)	0.0120	57
5 % H_2_/He, 423 K	4.7(4)	2.64(1)	0.0135	67
5 % CO/He, 423 K	3.5(3)	2.69(1)	0.0135	62
5 % H_2_/He, 473 K	5.7(4)	2.64(1)	0.0150	52
5 % CO/He, 473 K	5.2(3)	2.66(1)	0.0150	44
5 % H_2_/He, 573 K	5.1(4)	2.64(1)	0.0175	66
5 % CO/He, 573 K	3.5(4)	2.67(1)	0.0175	60

[a] Data range used was 3–11 Å^−1^; *R* fitting range was 1–6 Å; AFAC=1. Values in parentheses are statistical errors generated in the EXCURV98 program.

**Table 4:** Structural and statistical data of the Rh–Rh shell derived from the analysis of Rh K-edge EXAFS over 4 wt % Rh/CeO_*x*_/Al_2_O_3_ (method II) at temperatures and conditions indicated^[a]^

Conditions	CN	*r* [Å]	DW [Å^2^]	*R* [%]
5 % H_2_/He, 323 K	8.3(5)	2.66(1)	0.0115	57
5 % CO/He, 323 K	6.7(3)	2.67(1)	0.0115	47
5 % H_2_/He, 373 K	7.2(3)	2.65(1)	0.012	39
5 % CO/He, 373 K	6.2(3)	2.67(1)	0.012	43
5 % H_2_/He, 423 K	7.7(5)	2.64(1)	0.0135	64
5 % CO/He, 423 K	5.7(4)	2.65(1)	0.0135	59
5 % H_2_/He, 473 K	5.8(3)	2.64(1)	0.015	41
5 % CO/He at 473 K	4.7(3)	2.65(1)	0.015	58
5 % H_2_/He at 573 K	6.2(4)	2.64(1)	0.0175	52
5 % CO/He at 573 K	5.1(4)	2.66(1)	0.0175	60

[a] Data range used was 3–11 Å^−1^; *R* fitting range was 1–6 Å; AFAC=1. Values in parentheses are statistical errors generated in the EXCURV98 program.

In the case of 4 wt % Rh/CeO_*x*_/Al_2_O_3_ (method I), the Rh–Rh CN under 5 % H_2_/He is about 5.7 at 323 K and 4.7 at 423 K; these values correspond to about 16 and 10 atoms in the average spherical particle.[30] Rhodium particles of 4 wt % Rh/CeO_*x*_/Al_2_O_3_ (method II) under reducing conditions (<473 K) are clearly larger than non-ceriated and ceriated rhodium catalysts (method I; CN_Rh–Rh_≈8.3 at 323 K and 7.7 at 423 K). Assuming a regular (hemispherical) morphology with fcc structure,[30] an average particle would contain 68 atoms at 323 K and 37 atoms at 423 K. A similar trend was observed in fresh rhodium catalysts characterised by transmission electron microscopy (TEM).[34] A larger particle size distribution was observed in the TEM images for rhodium catalysts supported on ceria–alumina (method II), estimated as about 2.6 nm; in contrast to rhodium particles prepared with method I, which had an average size of about 1.9 nm. However, the particle size determined by TEM corresponded to both the metallic and oxidic fraction of the rhodium particles. On the other hand, similar structural behaviour for non-ceriated rhodium samples and rhodium catalysts doped with ceria (method I and II) throughout CO exposure emerged from the EDE analyses. The Rh–Rh bond length changes throughout the CO exposure, typically from 2.64 to about 2.67 Å, and there are also small changes in the CN for Rh–Rh (CN_Rh–Rh_) when the gas flow switches from He to CO. For both systems, the Rh particles are larger at the start of the experiment and they shrink during CO exposure, with the CN_Rh–Rh_ dropping by about 2.

**Figure 8:**
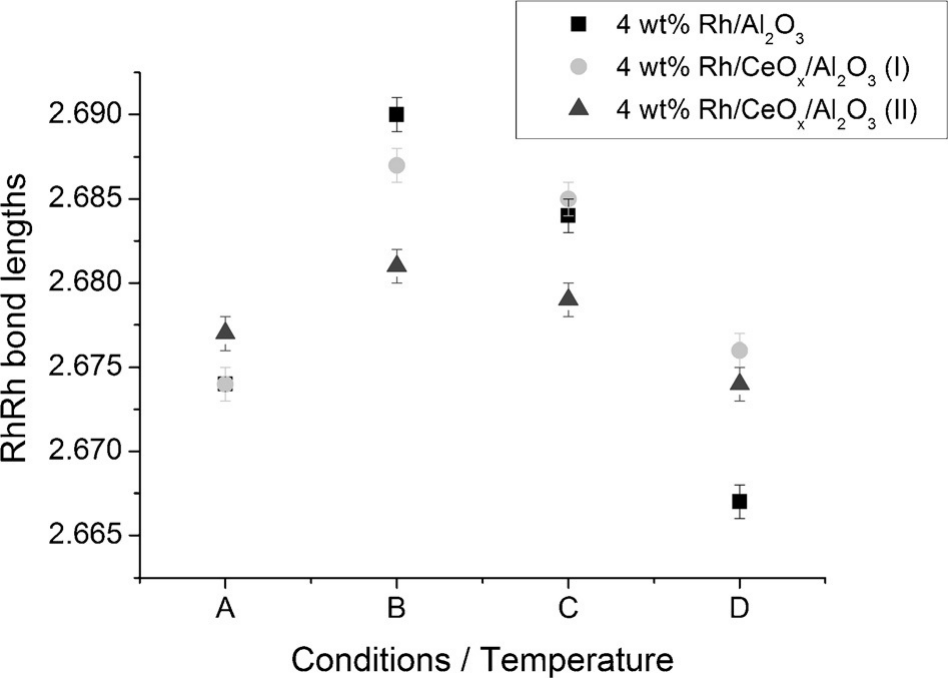
Variation of Rh–Rh bond lengths of 4 wt % Rh/Al_2_O_3_ and 4 wt % Rh/CeO_*x*_/Al_2_O_3_ (methods I and II) under different conditions: A) 5 % H_2_/He, 323 K; B) 5 % CO/He, 323 K; C) 5 % CO/He, 423 K; and D) 5 % CO/He, 573 K.

**Figure 9:**
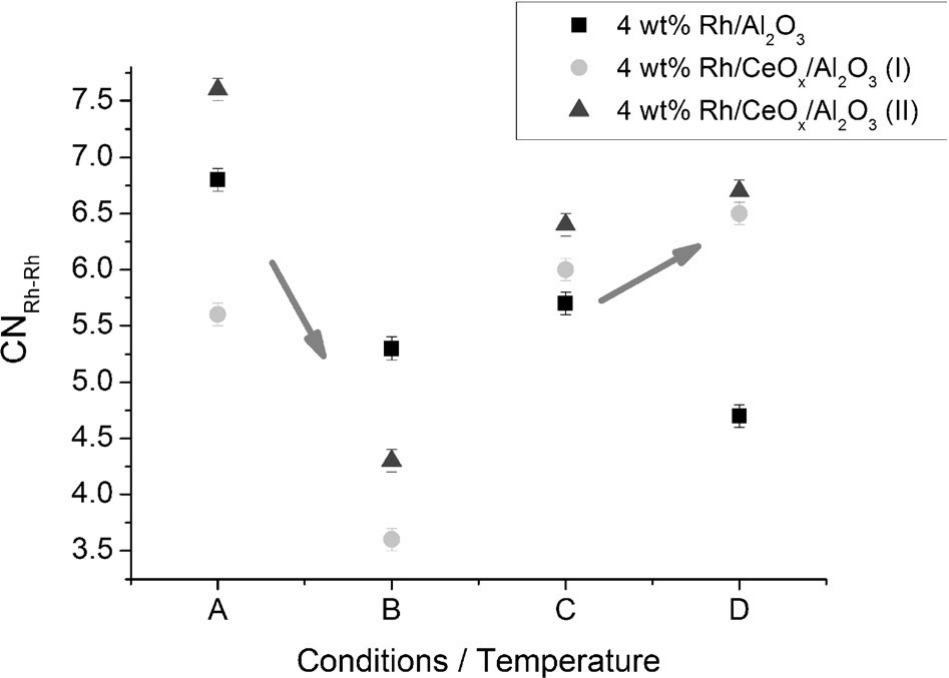
Variation of Rh–Rh CN of 4 wt % Rh/Al_2_O_3_ and 4 wt % Rh/CeO_*x*_/Al_2_O_3_ (methods I and II) under different conditions: A) 5 % H_2_/He, 323 K; B) 5 % CO/He, 323 K; C) 5 % CO/He, 423 K; and D) 5 % CO/He, 573 K.

Structural changes for the Rh particles before and after CO exposure at various temperatures were also monitored with scanning Rh K-edge XAFS. Variations of the Rh–Rh bond lengths and CN of non-ceriated and ceriated rhodium catalysts under different conditions (labelled A to D) are shown in Figures 8 and 9. Generally, comparable trends in structural changes after CO exposure were observed for each rhodium catalyst. Under H_2_/He (condition A), an identical Rh–Rh distance of 2.67 Å for Rh/Al_2_O_3_ and Rh/CeO_*x*_/Al_2_O_3_ catalysts was determined. The Rh–Rh CN determined by scanning EXAFS was in good agreement with that of the EDE results, for example, in the case of 4 wt % Rh/CeO_*x*_/Al_2_O_3_ (method I), CN_EXAFS_≈5.6 and CN_EDE_≈5.7. After CO exposure at 323 K (condition B), the Rh–Rh CN decreased, accompanied by elongation of the Rh–Rh bond lengths. The Rh–Rh bond lengths of ceriated rhodium catalysts were almost constant, with an increasing CN_Rh–Rh_ at an elevated temperature of 423 K (C). Increasing the temperature further to 573 K (condition D) decreased the Rh–Rh distance in all samples. The Rh–Rh CN, however, increased for ceriated rhodium catalysts and decreased for the non-ceriated rhodium catalyst. A smaller variation in CN_Rh–Rh_ throughout the whole experiment was observed for 4 wt % Rh/Al_2_O_3_ compared with that for ceriated rhodium catalysts. Variations in Rh–Rh CN as a function of temperature for ceriated Rh/Al_2_O_3_ determined by the EDE measurement are presented in Figure S8 in the Supporting Information. Because the CNs of Rh–Rh shell for the starting Rh catalysts (He, 323 K) collected by EDE and scanning XAS are comparable, discrepancies in the values of CN_Rh–Rh_ can be observed throughout CO exposure. After an initial drop of CN_Rh–Rh_ when exposed to CO at 323 K, a subsequent increase in the Rh–Rh contribution at higher temperature (423–573 K) is detected by scanning XAS, which is not the case of EDE measurements. This phenomenon can be explained by longer CO exposure to Rh catalysts within the scanning XAS measurement, for which an agglomeration of Rh particles takes place. The steady-state measurements give a valuable insight into the catalyst; however, dynamic EDE measurements will provide a more realistic demonstration of the studied system. Therefore, variations in structural changes as studied by these two spectroscopic approaches are expected.

**Figure 10:**
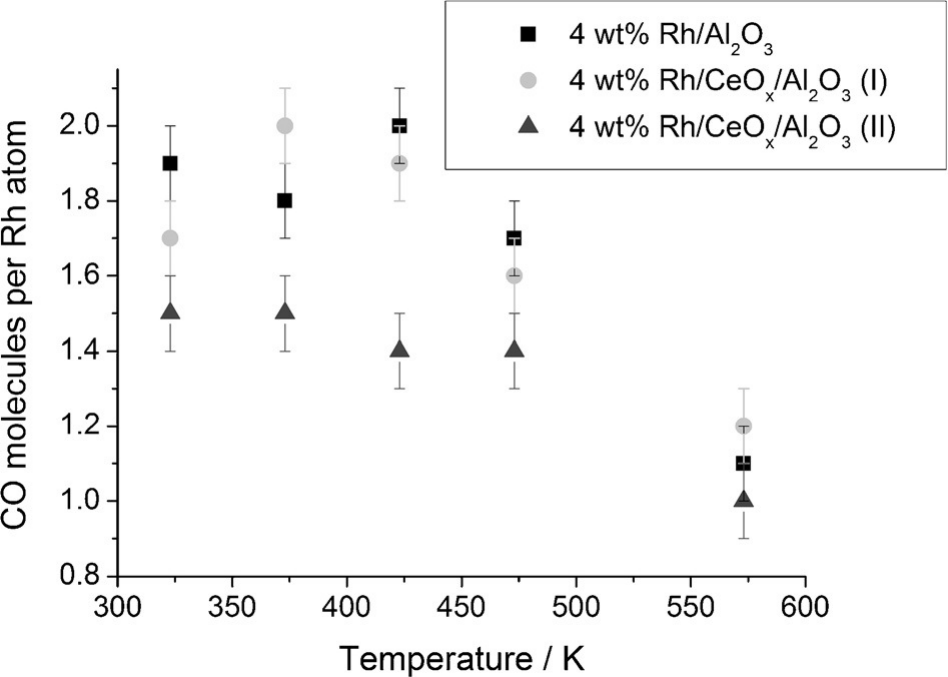
CO coverage for 4 wt % Rh systems as a function of temperature.

Generally, a decrease in CO uptake with temperature is observed (Figure 10), especially at 573 K, which supports the EXAFS/DRIFTS analysis. These results suggest either the agglomeration of Rh particles or no significant disruption of Rh particles upon CO exposure. In the temperature range of 323–423 K, approximately two CO molecules per Rh were adsorbed for Rh/Al_2_O_3_; this is quite high when considering the formation of geminal dicarbonyl species alongside the linear and bridged CO species. Moreover, EXAFS data indicate the presence of larger Rh particles, even after long CO exposure, for which not all Rh atoms are accessible for coordination to CO. Rhodium carbonyl clusters, such as Rh_6_(CO)_16_, have a higher Rh/CO ratio;[35] however, there has been no confirmation by other techniques that these species are present in the studied Rh catalyst. At higher temperatures, the CO uptake diminished, as confirmed by mostly linear and bridged species present on the Rh surface. Similar levels of CO uptake were observed for 4 wt % Rh/CeO_*x*_/Al_2_O_3_ (method I). However, a significantly lower CO uptake was measured for the ceriated rhodium catalyst prepared by method II, over the whole temperature range investigated. This is in good agreement with IR spectroscopy data, in which mostly the adsorption of linear CO species was observed.

**Figure 11:**
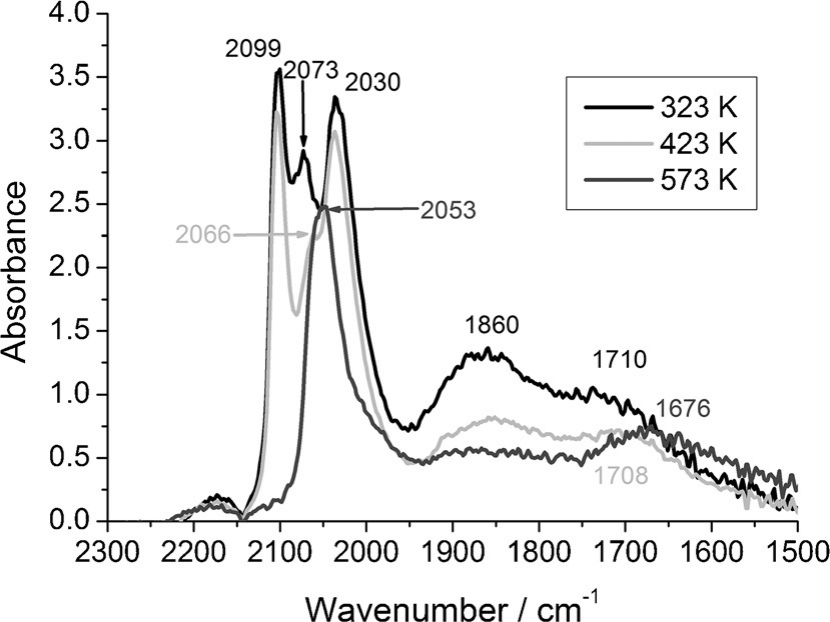
DRIFTS spectra of 1.6 wt % Rh/CeO_*x*_/Al_2_O_3_ prepared by method I after CO exposure at temperatures of 323, 423 and 573 K.

**Figure 12:**
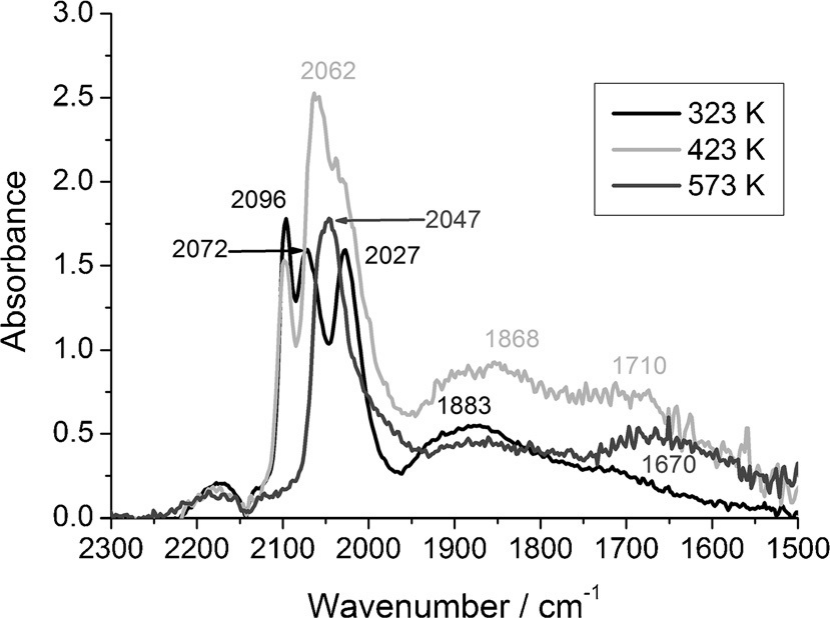
DRIFTS spectra of 1.6 wt % Rh/CeO_*x*_/Al_2_O_3_ prepared by method II after CO exposure at temperatures of 323, 423 and 573 K.

The effect of ceria was also studied over rhodium catalysts with lower rhodium loadings (1.6 wt % Rh). XAS results can be found in Figure S9 in the Supporting Information. Substantial oxidative disruption of Rh particles occurred for both ceriated Rh samples (method I and II), as highlighted by the decrease in the Rh–Rh CN throughout CO exposure. The variation of CN_Rh–Rh_ upon 5 % CO/He exposure indicates larger Rh particles were present in the ceriated Rh catalysts prepared by method II (e.g. ≈23 Rh atoms under He at 323 K), as compared with those prepared by method I (≈8 Rh atoms). No Rh–O shell was detected through scanning XAS measurements; however, EDE results clearly indicated the presence of a Rh–O contribution, which increased with temperature (Figure S7 in the Supporting Information). The lowest Rh–O CN is observed for ceriated Rh catalysts (method II) because of the present of the largest Rh particles detected by scanning XAS measurements. Furthermore, DRIFTS data (Figures 11 and 12) demonstrated that all three IR-active CO species formed during CO exposure, similar to that for undoped 1.6 wt % Rh/Al_2_O_3_. Larger proportions of the linear CO entities than other adsorbed CO species were formed at 323 K, compared with Rh/Al_2_O_3_. A blueshift of the linear CO band was also observed over both ceriated rhodium catalysts. Moreover, the presence of an additional band in the region of 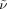
=1700 cm^−1^ for both samples indicates the close contact of rhodium and cerium atoms. A scanning transmission electron microscopy/energy-dispersive X-ray spectroscopy (STEM-EDX) study performed over a fresh form of these catalysts showed that rhodium particles were co-located with cerium atoms for both cerium-doped rhodium catalysts.[34] Furthermore, a X-ray photoelectron spectroscopy/X-ray absorption near-edge structure (XPS/XANES) study over fresh rhodium catalysts indicated that ceria located on the rhodium surface of Rh/CeO_*x*_/Al_2_O_3_ (method II) only provided partial protection for rhodium from oxidation. Therefore, it can be suggested that the pre-treatment procedure modifies the morphology of the Rh/Ce particles.

Compared with 4 wt % Rh/Al_2_O_3_, both ceriated Rh samples display a blueshift of linear CO frequency, with a larger variation of *ν*(CO) for the Ce-doped Rh catalyst (method II). This phenomenon is associated with larger rhodium particles and rhodium–cerium interactions. The electron density, which could be used to back bond to CO, is reduced and this consequently weakens the Rh–CO bond.[36] This was consistent with EXAFS results, in which the CN of the Rh–Rh shell was higher for the ceriated sample produced by method II than that for undoped Rh catalyst. Thus, cerium doping in this catalyst facilitates the protection of rhodium particles against facile oxidation and disruption.

### 2.3. Rh/CeO_*x*_/ZrO_2_/Al_2_O_3_

The effect of zirconia and ceria/zirconia on the structural behaviour of rhodium catalysts was studied with three samples with different Ce/Zr ratios, 0:1, 1:1 and 2:1, prepared by the CSM procedure (method II).

The DRIFTS results of the Zr and Zr-/Ce-doped Rh catalysts (Figure S10 in the Supporting Information) displayed the same three carbonyl-containing species [Rh–CO, Rh(CO)_2_ and bridged CO] formed on the rhodium surface of zirconia and ceria–zirconia-doped catalysts, analogously to the 4 wt % Rh/Al_2_O_3_ case. A redshift in the frequency of the linear CO species was observed to vary from 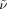
≈2067 cm^−1^ at 323 K to 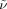
≈2039 cm^−1^ at 573 K for Rh/ZrO_2_/Al_2_O_3_. There was no peak at 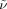
≈1700 cm^−1^ in the zirconiated-only sample, but this was evident in the mixed zirconium–cerium samples. The redshift for the linear CO species was also present for Ce-/Zr-promoted Rh catalysts; however, the position of this absorption occurred at higher frequency with increasing Ce loading. This phenomenon can be explained by the increased Ce interactions with Rh atoms, which reduces the electron density available for back bonding to adsorbed CO.[36] The relative amount of linear and bridged species compared with geminal dicarbonyl species increases with higher cerium doping in the rhodium system over the entire temperature range studied, which suggests stronger rhodium–cerium interactions. Furthermore, the blueshift of linear CO species increases with CeO_*x*_ doping. It has been previously shown that the introduction of ZrO_2_ into the CeO_*x*_ lattice generates structural modification of the Rh catalysts to enhance the redox properties of Ce^3+^/Ce^4+^ during the catalytic process.[37–39]

The EDE results show that zirconium-promoted rhodium catalysts (Figure S11 in the Supporting Information) have the largest value of CN_Rh−Rh_, which indicates the largest rhodium particle size (an average of 53 Rh atoms at 323 K) amongst the rhodium catalysts studied. Upon CO exposure at 323 K, the CN value for Rh–Rh seems to be constant; however, at elevated temperatures of 423–573 K, a decrease in CN_Rh–Rh_ of about 1 can be observed, which is barely significant. The incorporation of CeO_*x*_ into the zirconiated rhodium catalysts reduced the CN of Rh–Rh under all conditions investigated.

**Figure 13:**
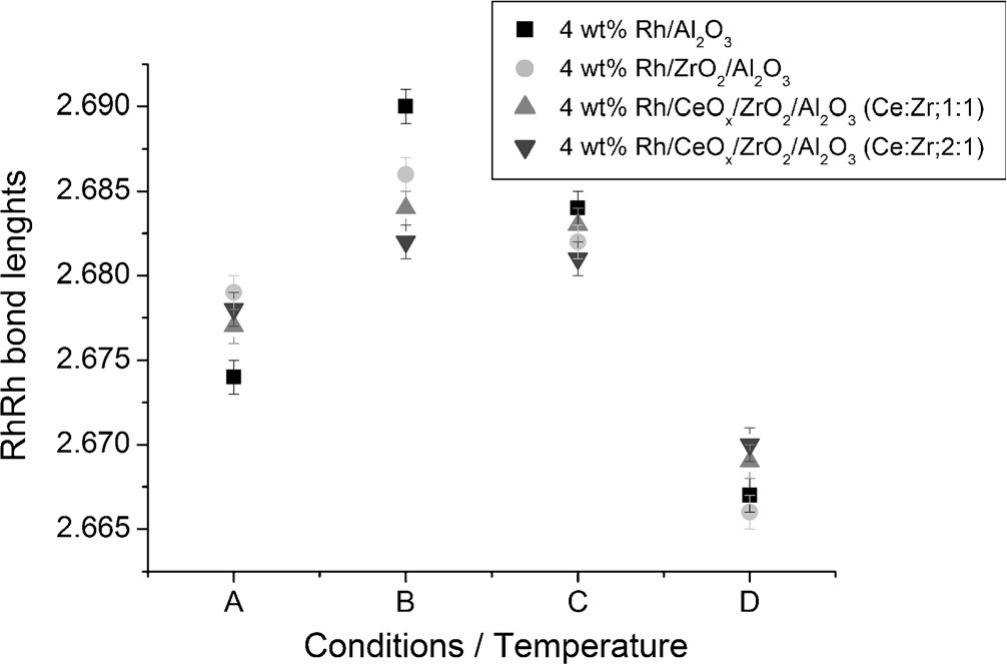
Variation of Rh–Rh bond lengths of 4 wt % Rh/Al_2_O_3_ and Ce-/Zr-modified Rh catalysts under different conditions: A) 5 % H_2_/He, 323 K; B) 5 % CO/He, 323 K; C) 5 % CO/He, 423 K; and D) 5 % CO/He, 573 K.

**Figure 14:**
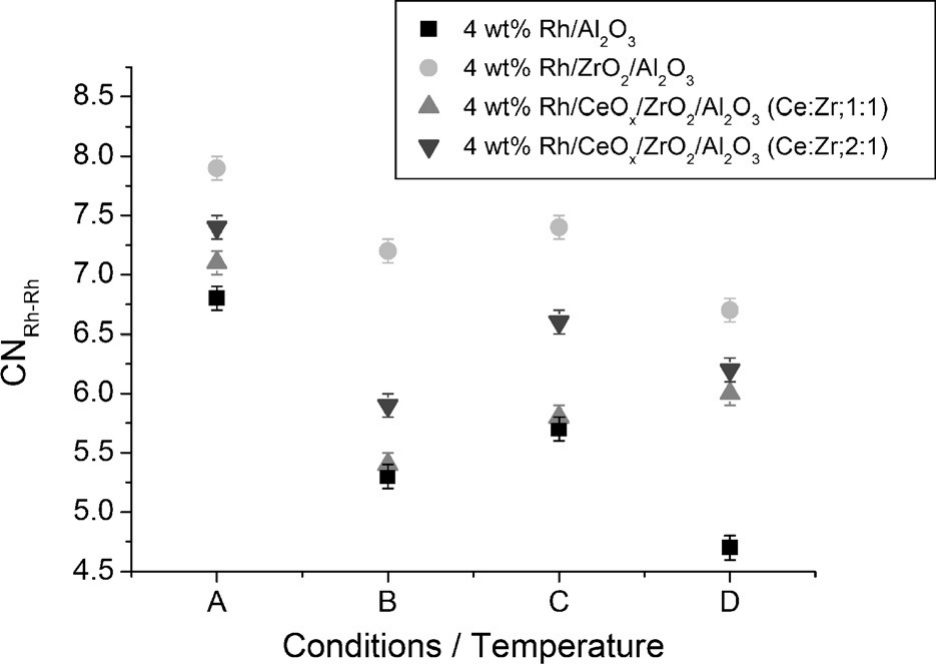
Variation of Rh–Rh CN for 4 wt % Rh/Al_2_O_3_ and Ce-/Zr-modified Rh catalysts under different conditions: A) 5 % H_2_/He, 323 K; B) 5 % CO/He, 323 K; C) 5 % CO/He, 423 K; and D) 5 % CO/He, 573 K.

Complementary scanning EXAFS studies (Figures 13 and 14) highlighted the structural changes to the rhodium particles during extensive exposure to carbon monoxide at different temperatures. A similar tendency in Rh–Rh bond lengths and CN_Rh–Rh_ variations as those for the reference Rh/Al_2_O_3_ system was observed. The increase in Rh–Rh distance, and reduction in Rh–Rh coordination, after CO exposure at 323 K, indicates that the Rh particles are eroded into smaller entities. The observed results correlate well with EDE data because similar trends in structural changes can be identified for each zirconium and cerium-/zirconium-doped rhodium catalysts. However, this phenomenon of Rh–Rh bond length expansion as the particles get smaller is not typical and cannot be easily explained. The general behaviour observed for “clean” metal nanoparticle results in a bond length contraction. Similar catalytic systems, such as Pd/Al_2_O_3_, were studied by the Freund group, wherein they measured lattice parameters as a function of particle size by TEM and reported a reduction of the distances within the lattice with decreasing cluster size.[40] Moreover, a recent study of γ-Al_2_O_3_-supported Pt particles with an average diameter of 1 nm demonstrated a Pt–Pt bond length contraction at elevated temperatures.[41] The largest rhodium particles were observed for zirconiated rhodium catalysts throughout CO exposure at the temperatures studied. These results suggest that Zr species are in intimate contact with Rh entities to form larger Rh particles under H_2_/He at 323 K, which consequently protects them against oxidative disruption upon exposure to CO/He. Our previous XAS studies showed that a zirconium-doped rhodium catalyst has a predominance of metallic Rh–Rh interactions when the catalyst is exposed to ambient conditions. On the other hand, TEM measurements indicated that this material yielded particle size distributions between 1.5 and 3 nm; these values are comparable to those of the non-promoted catalysts.[34]

The carbon monoxide uptake of zirconia and ceria–zirconia-doped rhodium catalysts (Figure S12 in the Supporting Information) changed little with increasing temperature. Amongst all Rh systems investigated, the lowest value of CO uptake at all temperatures (≈1.3 CO per Rh) was observed for 4 wt % Rh/ZrO_2_/Al_2_O_3_; this can be explained by the limited adsorption of CO on the extended Rh surface, because the EDE data indicates that these are the largest Rh particles of the systems investigated. This was also previously observed for in ceria-doped rhodium catalysts (method II). Ceria does not have the same effect on the CO coverage when doped with zirconia, it was detected in only ceriated rhodium catalysts (Figure 10), which would suggest that Ce-Zr provides additional sites for CO adsorption.

The DRIFTS spectra of 1.6 wt % rhodium catalysts doped with zirconia and ceria–zirconia (Figure S13 in the Supporting Information), exhibited the three main peaks assigned to geminal dicarbonyl, linear and bridged carbon monoxide species. However, the quality of the IR spectroscopy data at the lower adsorption levels excluded the possibility of the detection of other CO species, such as bridged Rh–(CO)–Ce. The EXAFS studies of zirconia and ceria–zirconia-doped 1.6 wt % Rh catalysts (Figure S14 in the Supporting Information) indicated relatively large Rh particles, which were comparable to the size of those in the 4 wt % Rh/Al_2_O_3_ sample.

Herein, clear correlations are shown between the temperature and cerium/zirconium doping on the disruption of rhodium nanoparticles. The EXAFS results on undoped Rh catalyst clearly show the oxidative disruption of the Rh particles and the formation of isolated Rh^+^ sites, as indicated by the DRIFTS study during CO exposure up to 423 K. The evolution of Rh^I^(CO)_2_ species can therefore be directly correlated to the disruption of Rh particles. This result correlates well with previous findings of the disruptive capacity on similar rhodium systems, in which the corrosion of rhodium nanoparticles is associated with the adsorption of geminal dicarbonyl species.[5, 7, 42] Distinct structural–functional differences were observed for ceriated rhodium catalysts, which depended on the synthetic procedure. The initial Rh particle size for 4 wt % Rh/CeO_*x*_/Al_2_O_3_ (method I) is much smaller than that of ceriated Rh catalysts produced by method II. Upon exposing ceriated Rh catalysts to 5 wt % CO/He, the EDE data indicates that a similar level of Rh disruption occurs on the Rh surface at various temperatures. For Rh/CeO_*x*_/Al_2_O_3_ (method I), smaller Rh particles exist throughout CO exposure. On the other hand, the IR spectroscopy and MS results highlight that similar adsorption characteristics and overall processes are involved. Larger rhodium particles of ceriated rhodium catalyst (method II) were detected during the entire carbon monoxide exposure compared with those of ceria-doped rhodium catalysts (method I). As proposed in our study, ceria in this sample is in intimate contact with the rhodium particles, isolating them from facile oxidation. An XPS study reported by Suopanki et al. indicated that the fraction of reducible rhodium had the best catalytic activity; they explained the existence of metallic rhodium in the system by burying of the active material into the bulk of the washcoat.[43] The absence of the geminal dicarbonyl and the adsorption of mostly linear and bridged CO species on metallic Rh also indicate the proximity of Rh and Ce species. These CO sites are in relatively good agreement with those reported for CO adsorbed on Rh/reduced CeO_2_ (111) monitored by high-resolution electron energy loss (HREEL) spectroscopy.[44] The additional bridged CO species adsorbed between Rh and Ce atoms for all ceriated Rh catalysts clearly highlights their proximity, as indicated by the STEM EDX high-angle annular dark field (HAADF) line profile analysis in our previous studies.[34]

Incorporation of ZrO_2_ in the undoped and ceriated Rh catalysts increases the Rh–Rh occupation under conditions investigated. Zirconium-doped Rh/Al_2_O_3_ exhibits the largest Rh particles among all Rh samples investigated. The lowest CO update observed can be explained by the steric and electronic limitations of the extended Rh surface, as previously observed in ceriated Rh catalysts (method II). However, the adsorption of geminal dicarbonyl, linear and bridged CO species was demonstrated by DRIFTS; this suggested that Zr did not protect the Rh particles in the same way as that of Ce doping, even with the same preparation method. No IR signal was detected in the 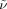
=1700–1200 cm^−1^ region previously ascribed to carbonates linked to zirconia; this can be attributed to a low ZrO_2_ loading in the systems investigated.[45]

## 3. Conclusions

Carbon monoxide interactions with a variety of ceriated and zirconiated rhodium catalysts were investigated by using an array of DRIFTS/EDE/MS techniques in a time-resolved, in situ manner.

Generally, in the range of temperatures from 323 to 423 K, the IR spectroscopy results after CO exposure were dominated by geminal dicarbonyl species adsorbed on Rh, whereas EXAFS analysis suggested the reduction of Rh particle size and elongation of Rh–Rh bonds. The most significant changes were observed for rhodium catalysts doped by ceria, which were synthesised by the CSM method (method II). Ceria deposited on pre-supported rhodium catalysts reduced Rh-γ-Al_2_O_3_ interactions and, as a result, CO was mainly adsorbed as linear and bridged species on the surface of supported rhodium. Rhodium catalysts with zirconium doping contained the largest rhodium particles, but still afforded geminal dicarbonyl, linear and bridged CO species. However, they exhibited the lowest CO uptake. The addition of CeO_*x*_ in combination with ZrO_2_ decreased the CN of Rh–Rh and larger proportions of linear and bridged CO species were adsorbed on the Rh surface; this was attributed to ceria offering protection to rhodium from facile oxidation. However, in our DRIFTS/XAS/MS studies of ceria–zirconia-promoted Rh catalysts, no particular ZrO_2_ effect on the behaviour of CeO_*x*_ could be detected. Only the cerium-containing samples displayed the low-frequency *ν*(CO) band, in which a Ce^*n*+^ site appeared to act as a Lewis acid, which weakened the bridging CO group on a rhodium particle. This, in addition to oxygen storage, may influence the catalytic activity of such materials.

## Experimental Section

### Preparation of Rh/γ-Al_2_O_3_

The 4 wt % Rh/γ-Al_2_O_3_ supported samples were prepared through wet impregnation of Al_2_O_3_ (Degussa, Alumina C, surface area ca. 88 m^2^ g^−1^; 1.92 g) with RhCl_3_**⋅**3 H_2_O (0.21 g) in aqueous solution. This was stirred, by using a Teflon-coated magnetic stirrer bar, until a uniform paste was achieved. The sample was then dried in air. Subsequently, the resultant sample was calcined for 6 h at 673 K in 5 % O_2_/He and reduced for 5 h under a flow of 5 % H_2_/He at 573 K.

### Preparation of Ceriated Rh/γ-Al_2_O_3_ (Method I)

The 5 wt % Ce/γ-Al_2_O_3_ support was produced by dissolving cerium(III) 2,4-pentanedionate (0.509 g) in toluene, to which γ-Al_2_O_3_ (1.805 g) was added. The sample was dried overnight in air before being calcined under 5 %O_2_/He for 6 h at 773 K. Subsequently, the solution of RhCl_3_**⋅**3H_2_O in water was added to a suspension of Al_2_O_3_/CeO_2_ support and stirred. The sample was then dried overnight in air before being calcined under 5 % O_2_/He for 6 h at 773 K and reduced under 5 % H_2_/He for 5 h at 300 °C.

### Preparation of Ceriated, Zirconiated and Ce/Zr Rh/γ-Al_2_O_3_ (Method II)

Rh/γ-Al_2_O_3_,( 1 g) was re-reduced under a flow of 5 % H_2_/He for 3 h at 573 K. To prepare Rh catalysts with 5 wt % Ce, a solution of [Ce(acac)_3_] (0.164 g) in toluene (100 mL) was placed in the three-way tap dropper, purged with N_2_ for 15 min and added dropwise to the reduced catalyst. Subsequently, the reagents were mixed under a flow of 5 % H_2_/He at 353 K for 8 h. Then, the sample was filtered and dried in air overnight. The sample was again reduced under 5 % H_2_/He at 573 K for 3 h. All preparations were performed under N_2_ atmosphere. To produce Rh catalysts doped with Zr, [Zr(acac)_4_] (0.281 g) was dissolved in toluene (100 mL). Catalysts promoted by ceria and zirconia at two different mixture ratios (Ce/Zr 1:1 or 2:1) were produced by simultaneously dissolving the appropriate amount of [Ce(acac)_3_] and [Zr(acac)_4_] in toluene.

Prior to XAFS measurements, all the samples were reduced in situ (in the DRIFTS cell). These samples were denoted “reduced”. The in situ reduction was performed as follows: 1) reduction in 5 % H_2_/He up to 573 K; 2) oxidation under 5 % O_2_/He at 573 K until remaining carbonaceous deposits were removed from the catalyst (by observing carbon-related fragments by MS); 3) at 573 K, back to 5 % H_2_/He; and 4) cooling to room temperature in 5 % H_2_/He.

### EDE

Rhodium K-edge XAFS spectra were measured in transmission mode by using a Si(311) polychromator mounted in a Bragg configuration. The EDE measurements were performed at the ESRF in Grenoble, France, at ID24. EXAFS detection was achieved through the FReLoN CCD camera with a total acquisition time for 10 spectra of about 10 ms. The DRIFTS measurements were performed simultaneously, with the same sampling rates for each spectroscopy, whilst a mass spectrometer continuously measured the composition of the gas phase.

### DRIFTS

The IR measurements were performed by using a Bruker IFS 66/S spectrometer equipped with a narrow band, linearised, MCT detector with 4 cm^−1^ resolution. The acquisition time for a single spectrum was about 100 ms.

### XAFS

Rhodium K-edge XAFS spectra were mainly measured by using a Si(311) monochromator at BM29 of the ESRF in Grenoble, France. The measurements were performed in transmission mode by using optimised ionisation chambers as detectors.

### Data Handling and Analysis

The EDE calibration was performed by using the XOP program[46] by comparing an EDE foil spectrum to an EXAFS foil spectrum and correcting the offset of the edge jump and multiplying the pixel number by a factor to obtain the energies. From the calibrated EDE foil, the offset and multiplication factor parameters were used to calibrate the EDE spectra collected. Data reduction was performed by using Xmult[47] and analysis was achieved by using a spherical wave formalism in the EXCURV98 program.[48] The *R* factors quoted herein were defined as *R*=(∫[*χT*−*χE*]*kn*d*k*/[*χE*]*kn*d*k*)×100 %, in which *χT* and *χE* were the theoretical and experimental EXAFS results, *k* was the photoelectron wave vector, d*k* was the range of photoelectron wave vectors analysed, and *n* was the weighting in *k* applied to the data. The number of parameters, *N*, that could be justifiably fit was estimated from the Nyqvist equation: *N*=(2Δ*k*Δ*r*/π)+1, in which Δ*k* and Δ*r* were the ranges in *k* and *r* space over which the data were analysed. DW factors for Rh–Rh and Rh–O shells were estimated for Rh/Al_2_O_3_, and subsequently, the spectra for the whole range of Rh catalysts were analysed in the same *k* range holding DW factor constant (2*σ*^2^=0.012 *σ*^2^). The error in CN should be considered in the range +/−10–20 %; errors in bond length determination were estimated at about 2 %. The value of *E*_f_ for each refinement was within the energy range of (−5) to 5 eV.

## Supporting Information

As a service to our authors and readers, this journal provides supporting information supplied by the authors. Such materials are peer reviewed and may be re-organized for online delivery, but are not copy-edited or typeset. Technical support issues arising from supporting information (other than missing files) should be addressed to the authors.

miscellaneous_information
