# Supplementary material for: Time-Resolved, In Situ DRIFTS/EDE/MS Studies on Alumina-Supported Rhodium Catalysts: Effects of Ceriation and Zirconiation on Rhodium–CO Interactions
Source: Chemphyschem. 2014 Jul 18;15(14):3049–59. doi: 10.1002/cphc.201402122 (PMC4529662; doi:10.1002/cphc.201402122)
Supplement: Supplementary file 1 — miscellaneous_information [file cphc0015-3049-sd1.pdf]

CHEMPHYSCHEM

Supporting Information

© Copyright Wiley-VCH Verlag GmbH & Co. KGaA, 69451 Weinheim, 2014

Time-Resolved, In Situ DRIFTS/EDE/MS Studies on Alumina-Supported Rhodium Catalysts: Effects of Ceriation and Zirconiation on Rhodium–CO Interactions**

Anna B. Kroner,[a, d] Mark A. Newton,*[b] Moniek Tromp,[c, d] Otello M. Roscioni,[d]
Andrea E. Russell,[d] Andrew J. Dent,[a] Carmelo Prestipino,[e] and John Evans*[a, d, f]

cphc_201402122_sm_miscellaneous_information.pdf

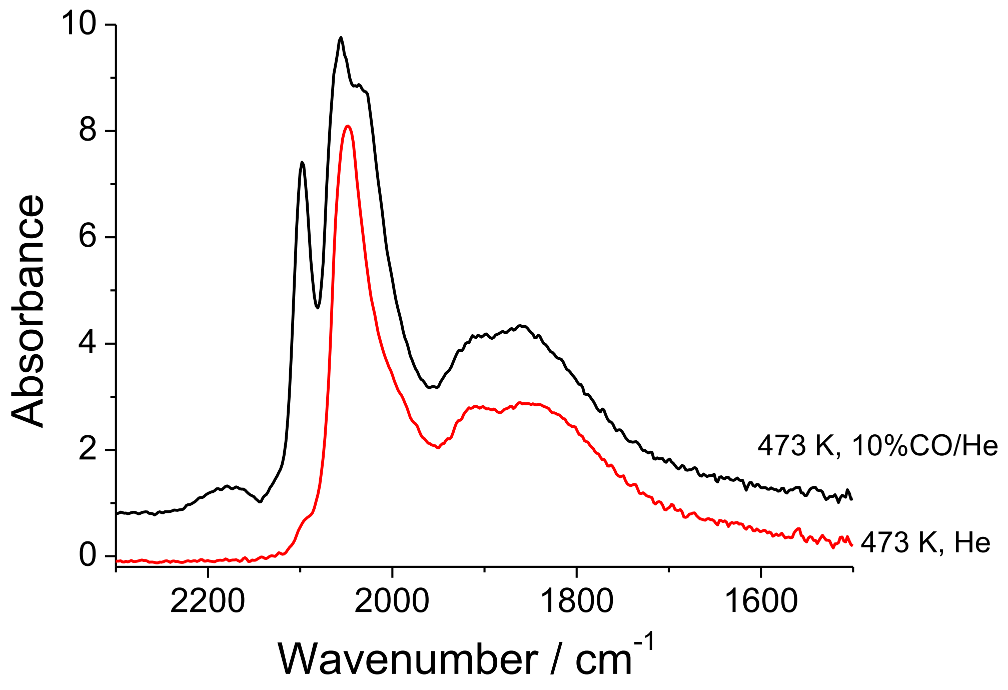

Figure S1. DRIFTS spectra of 4 wt% Rh/Al_2O_3 after CO exposure (black line) and subsequent He purge (red line) at 473 K.

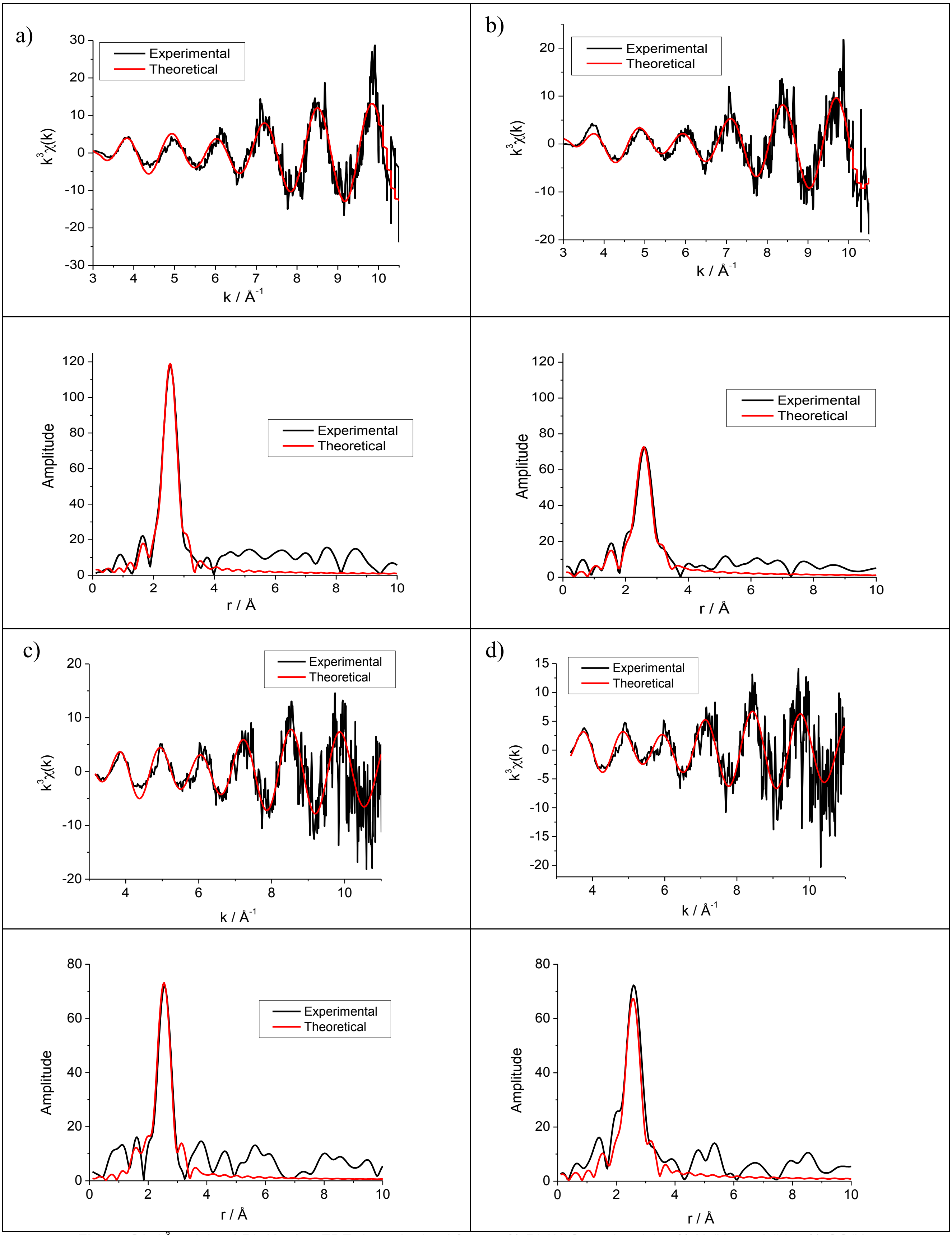

Figure S2. k^3 weighted Rh K edge EDE data obtained for 4 wt% Rh/Al_2O_3 under; (a) 5 % H_2/He and (b) 5 % CO/He (average of ten spectra taken at 59 s) at 323 K and under: (c) 5 % H_2/He and (d) 5 % CO/He at 573 K. The corresponding Fourier transforms are given underneath each spectrum.

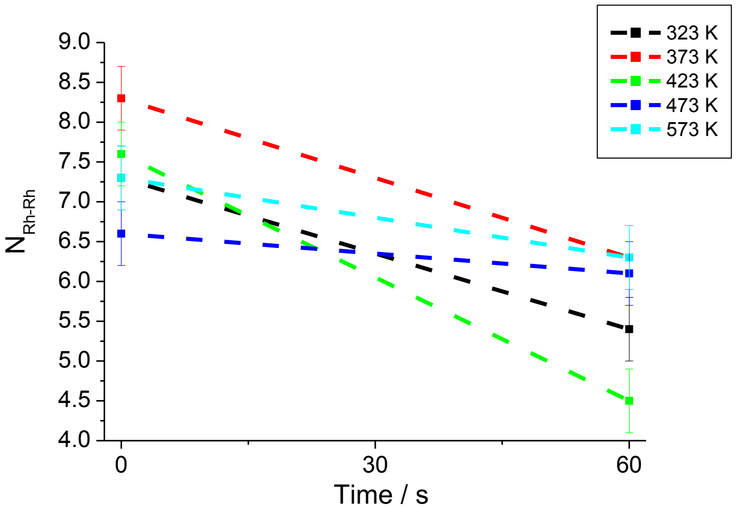

Figure S3. Variation of RhRh coordination number of 4 wt% Rh/Al_2O_3 before and after CO exposure as a function of temperature

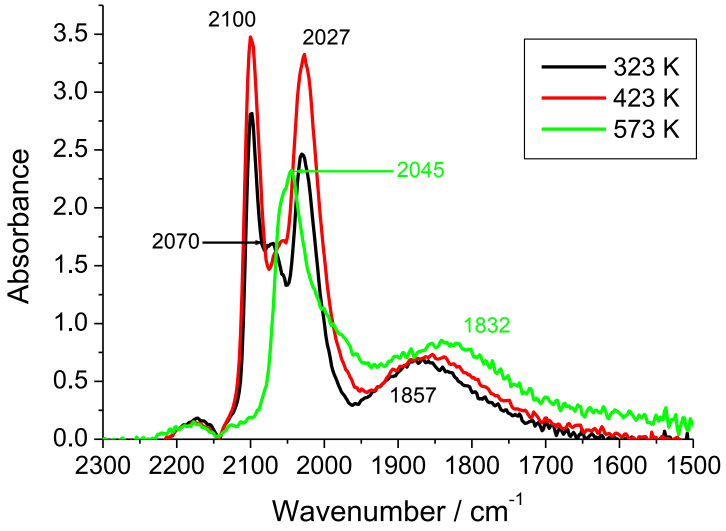

Figure S4. DRIFTS spectra of 1.6 wt% Rh/Al_2O_3 after CO exposure at three different temperatures: 323 K, 423 K, and 573 K

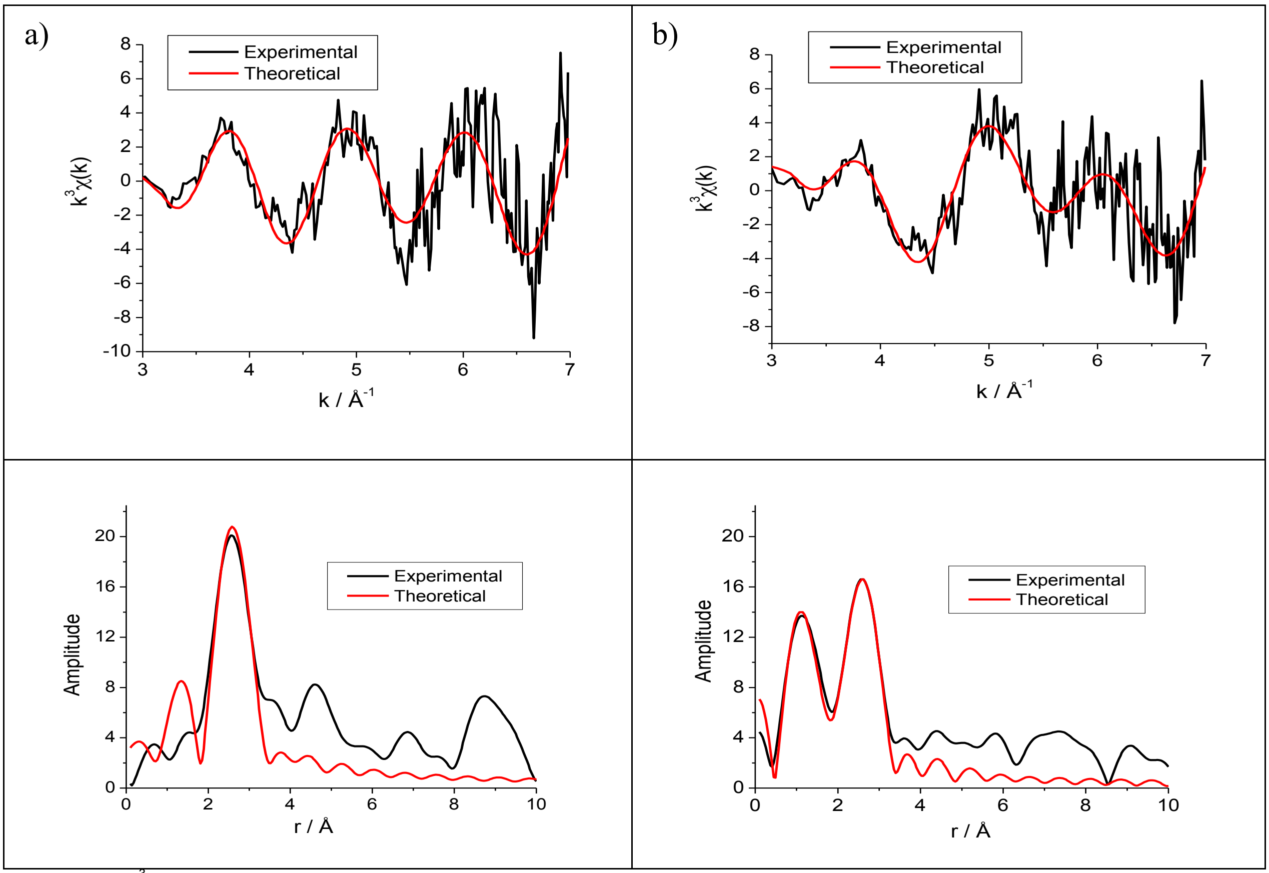

Figure S5. k^3 weighted Rh K edge EDE data obtained for 1.6 wt% Rh/Al_2O_3 under; (a) 5 % H_2/He and (b) 5 % CO/He (average of ten spectra taken at 59 s) at 323 K. The corresponding Fourier transforms are given underneath each spectrum.

Conditions	Scatterer	CN	r / Å	E_F	k (Å^{-1})	$2\sigma^2$/Å^2	R (%)
5 % H_2/He at 323 K	Rh	5.1 (5)	2.63 (1)	-2.3	3-7	0.0115	60
5 % CO/He at 323 K	Rh	3.6 (6)	2.63 (1)	-3.5	3-7	0.0115	55
	O	1.3 (3)	1.99 (3)			0.012	

Table S1. Structural and statistical data derived of 1.6 wt% Rh/Al_2O_3 from the analysis of spectra given in Figure S4

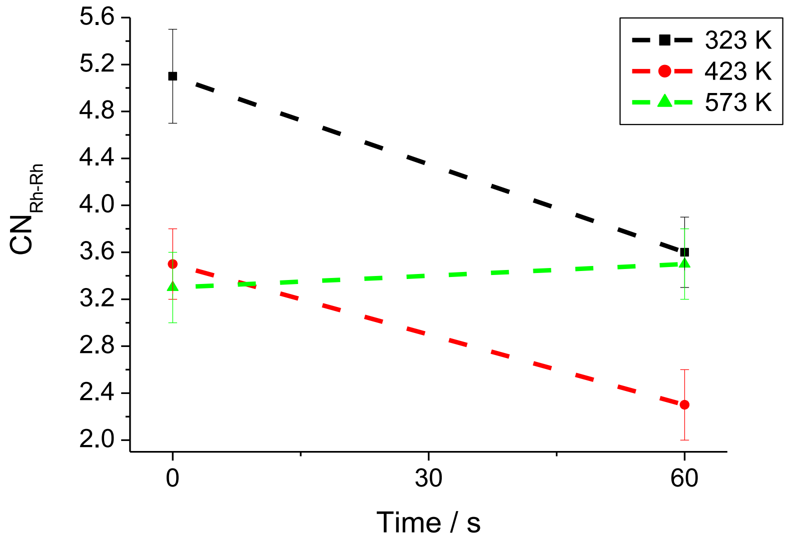

Figure S6. Variation of RhRh coordination number of 1.6 wt% Rh/Al_2O_3 before and after CO exposure as a function of temperature

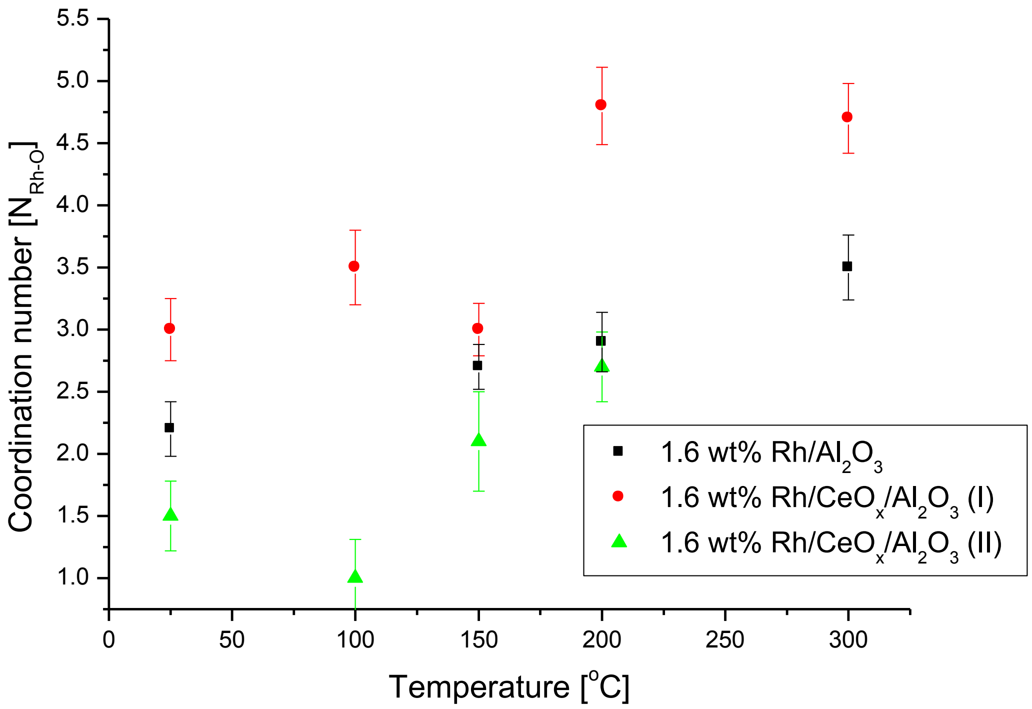

Figure S7. Variation of RhO coordination number of 1.6 wt% Rh catalysts as a function of temperature

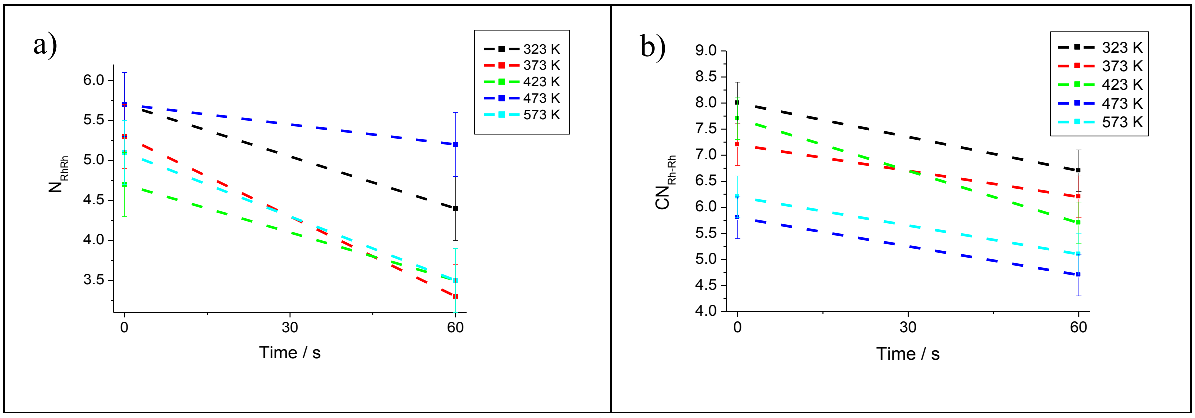

Figure S8. Variation of RhRh coordination number of 4 wt% Rh/CeO_x/Al_2O_3: a) method I, b) method II before and after CO exposure as a function of temperature

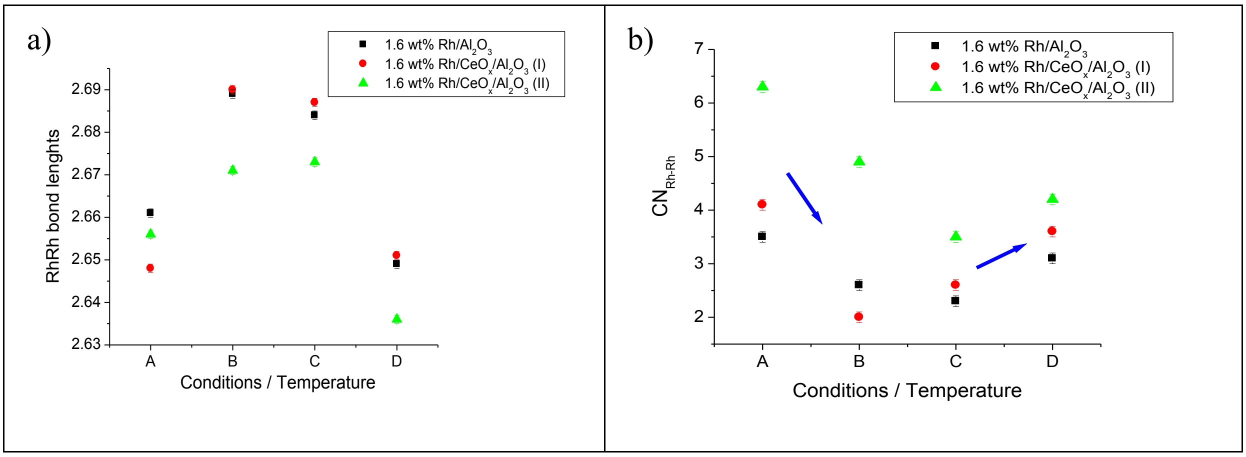

Figure S9. Variation of RhRh bond lengths (a) and RhRh coordination number (b) of 1.6 w% Rh/Al_2O_3 and 1.6 wt% Rh/CeO_x/Al_2O_3 (method I, II) under different conditions; A – 5 % H_2/He, 323 K, B – 5 % CO/He, 323 K, C – 5 % CO/He, 423 K, D – 5 % CO/He, 573 K.

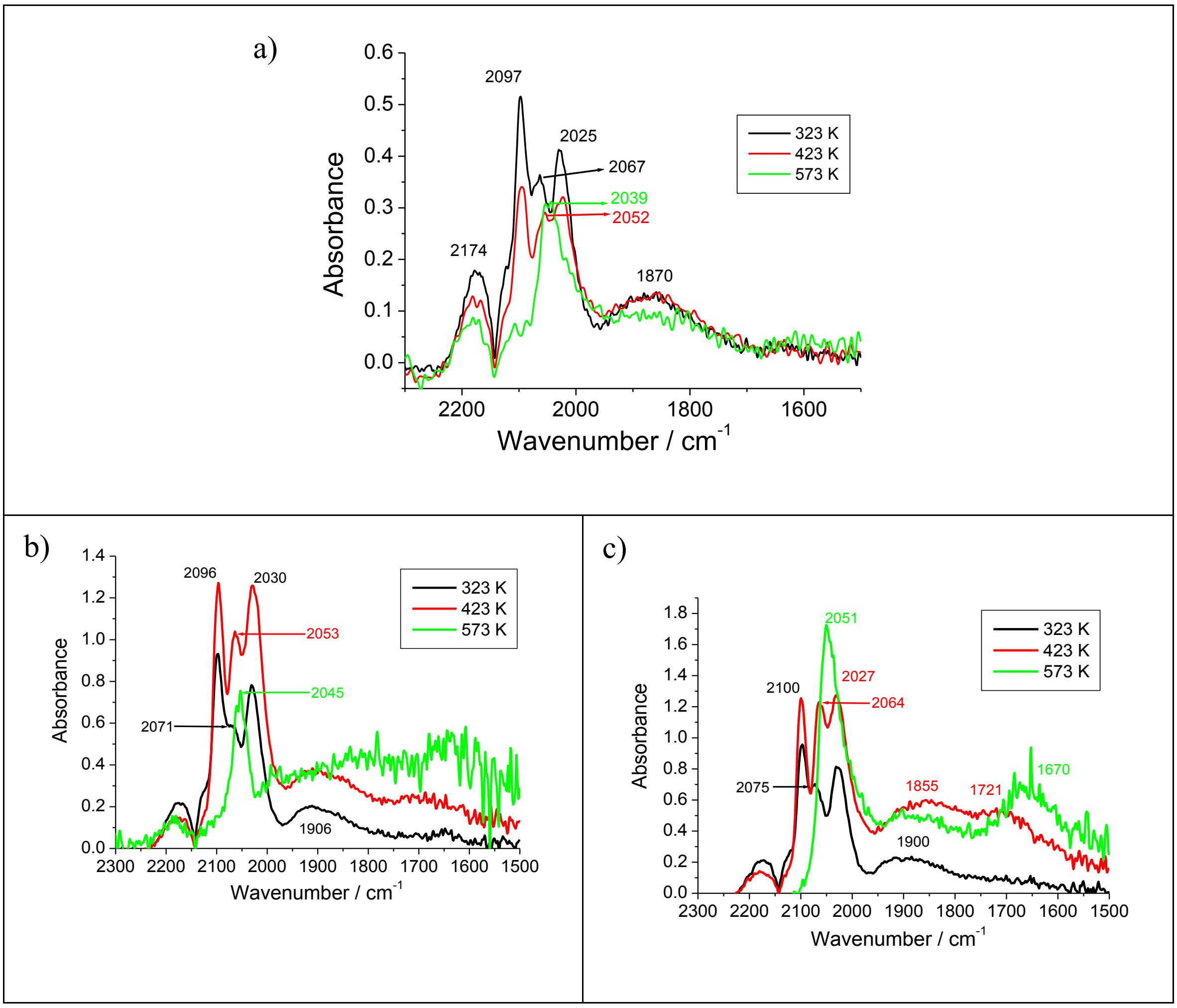

Figure S10. DRIFTS spectra of 4 wt% Rh/ZrO_2/Al_2O_3 (a), 4 wt% Rh/CeO_x/ZrO_2/Al_2O_3 (Ce:Zr;1:1); (b) (Ce:Zr;2:1) (c) after CO exposure at varies temperature: 323 K, 423K, 573 K

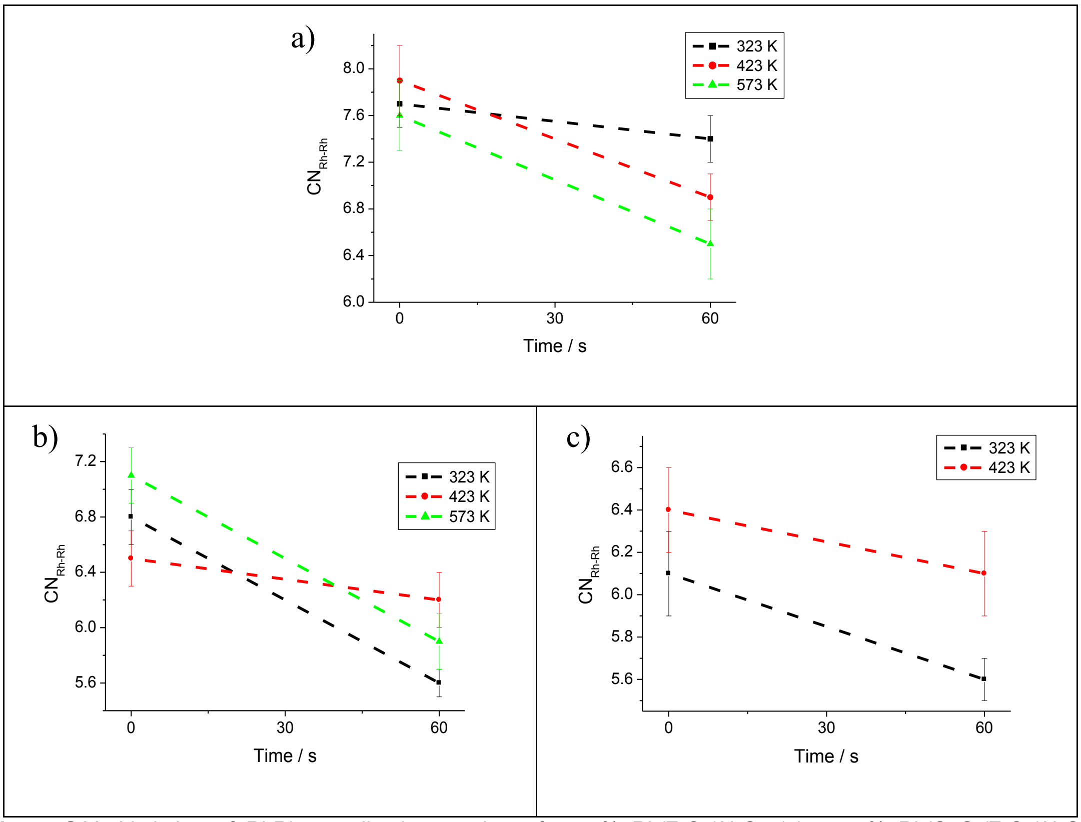

Figure S11. Variation of RhRh coordination number of 4 wt% Rh/ZrO_2/Al_2O_3 (a), 4 wt% Rh/CeO_x/ZrO_2/Al_2O_3, (Ce:Zr;1:1) (b); (Ce:Zr;2:1) (c) before and after CO exposure at the temperature indicated.

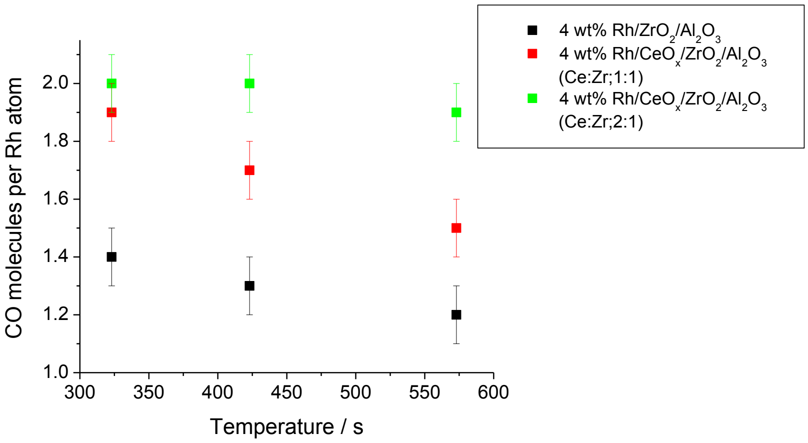

Figure S12. CO coverage for 4 wt% Rh systems in the function of temperature

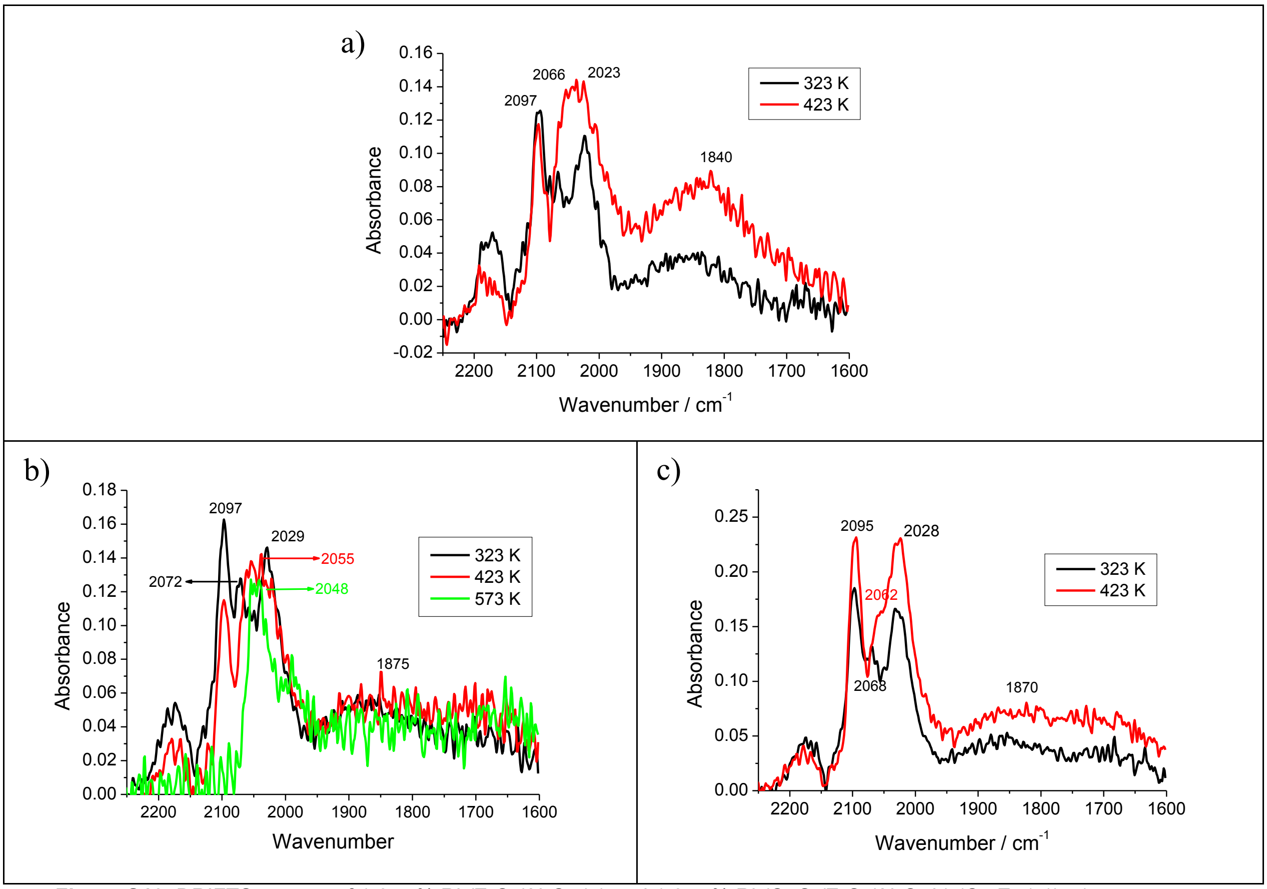

Figure S13. DRIFTS spectra of 1.6 wt% $Rh/ZrO_2/Al_2O_3$ (a) and 1.6 wt% $Rh/CeO_x/ZrO_2/Al_2O_3$ b) (Ce:Zr;1:1); c) (Ce:Zr;2:1) after CO exposure at varies temperature: 323 K, 423K, 573 K

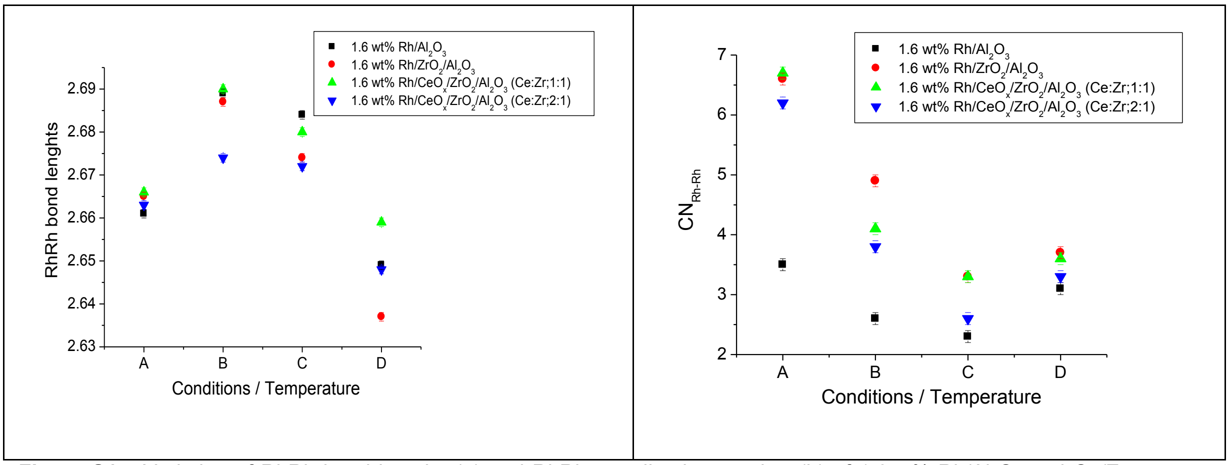

Figure S14. Variation of RhRh bond lengths (a) and RhRh coordination number (b) of 1.6 w% Rh/Al_2O_3 and Ce/Zr modified Rh catalysts under different conditions; A – 5 % H_2/He, 323 K, B – 5 % CO/He, 323 K, C – 5 % CO/He, 423 K, D – 5 % CO/He, 573 K.
